# Supplementary material for: Antisense oligonucleotide and adjuvant exercise therapy reverse fatigue in old mice with myotonic dystrophy
Source: Mol Ther Nucleic Acids. 2020 Nov 26;23:393–405. doi: 10.1016/j.omtn.2020.11.014 (PMC7787993; doi:10.1016/j.omtn.2020.11.014)
Supplement: Document S1. Figures S1–S6 and Tables S1 and S2 [file mmc1.pdf]

OMTN, Volume *23*

Supplemental Information

Antisense oligonucleotide and adjuvant exercise therapy reverse fatigue in old mice with myotonic dystrophy

Ningyan Hu, Eunjoo Kim, Layal Antoury, Jia Li, Paloma González-Pérez, Seward B. Rutkove, and Thurman M. Wheeler

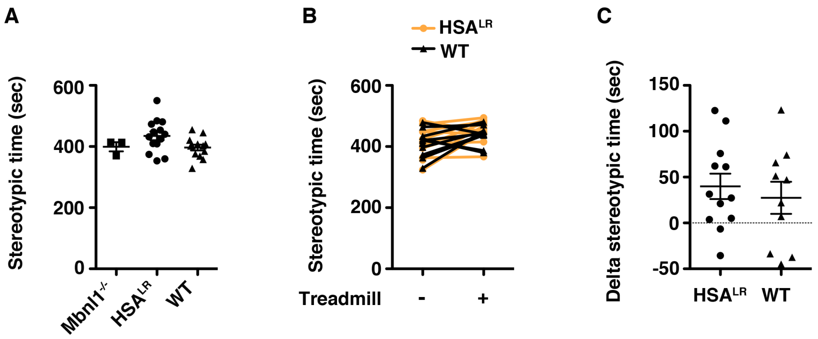

Figure S1. Spontaneous activity in two mouse models of DM1. We examined spontaneous x-, y-, and z-plane activity of untreated mice for 30 minutes in the dark either at night or in a reverse light cycle room, when mice are more active. (A) Quantitative activity measurements of the total time spent on small rapid movements such as scratching, grooming, or other stereotypic non-ambulatory movements (stereotypic time; seconds) during the 30 minute (1800-second) monitoring period in 2 - 3 month-old Mbnl1 knockout (-/-; N = 3), HSA^{LR} (N = 14), and wild-type (N = 13) immediately following treadmill exercise running for 10 minutes uphill at a 15 degree incline (see Materials and Methods). (B) Stereotypic time in 15 month-old HSA^{LR} (N = 12; orange circles) and WT (N = 10; black triangles) mice pre (-) and post (+) treadmill exercise running for 10 minutes uphill at a 15 degree incline. (C) The difference (delta) between the pre- and post-exercise measurements of each individual mouse shown in (B). Error bars indicate mean ± SEM. See also Figures 1 and 2.

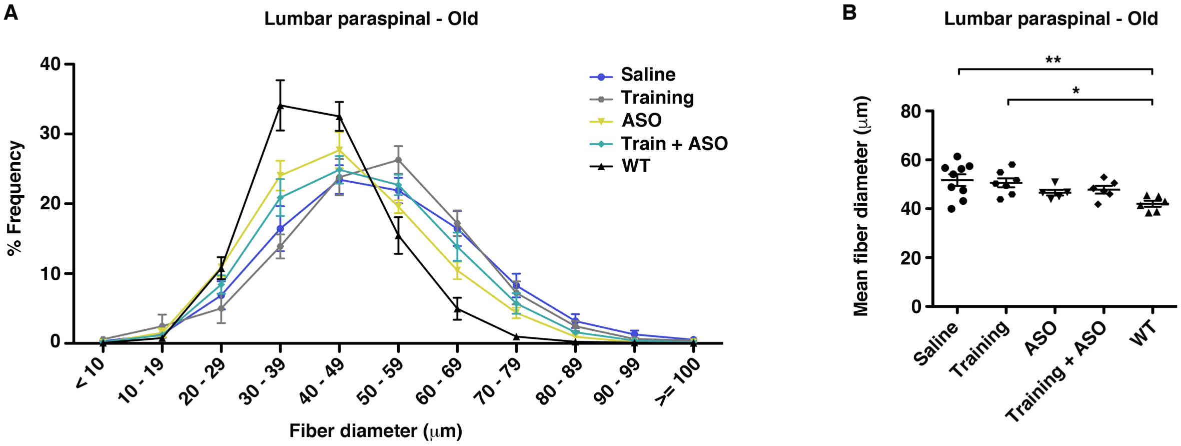

Figure S2. Relationship of fatigue in DM1 mice to muscle fiber diameter. We treated old HSA^{LR} mice (~ 14 months of age) with saline (N = 9), moderate intensity exercise training program (N = 7; Training; see Materials and Methods), ASO 445236 (N = 5), or a combination of training and ASO 445236 (N = 6; Train + ASO) for 3 ½ months and analyzed at ~ 18 months of age. Old wild type (WT; ~ 18 months of age) (N = 6) served as controls. (A) Minimum Feret's muscle fiber diameter, defined as the minimum distance between parallel tangents, in 8 *μ*m cryosections of lumbar paraspinal muscles in each group. (B) Mean fiber diameter in each group using the data in a). ** $P < 0.01$; * $P = 0.012$; one-way ANOVA/Tukey. Error bars indicate mean ± SEM. See also Figure 3.

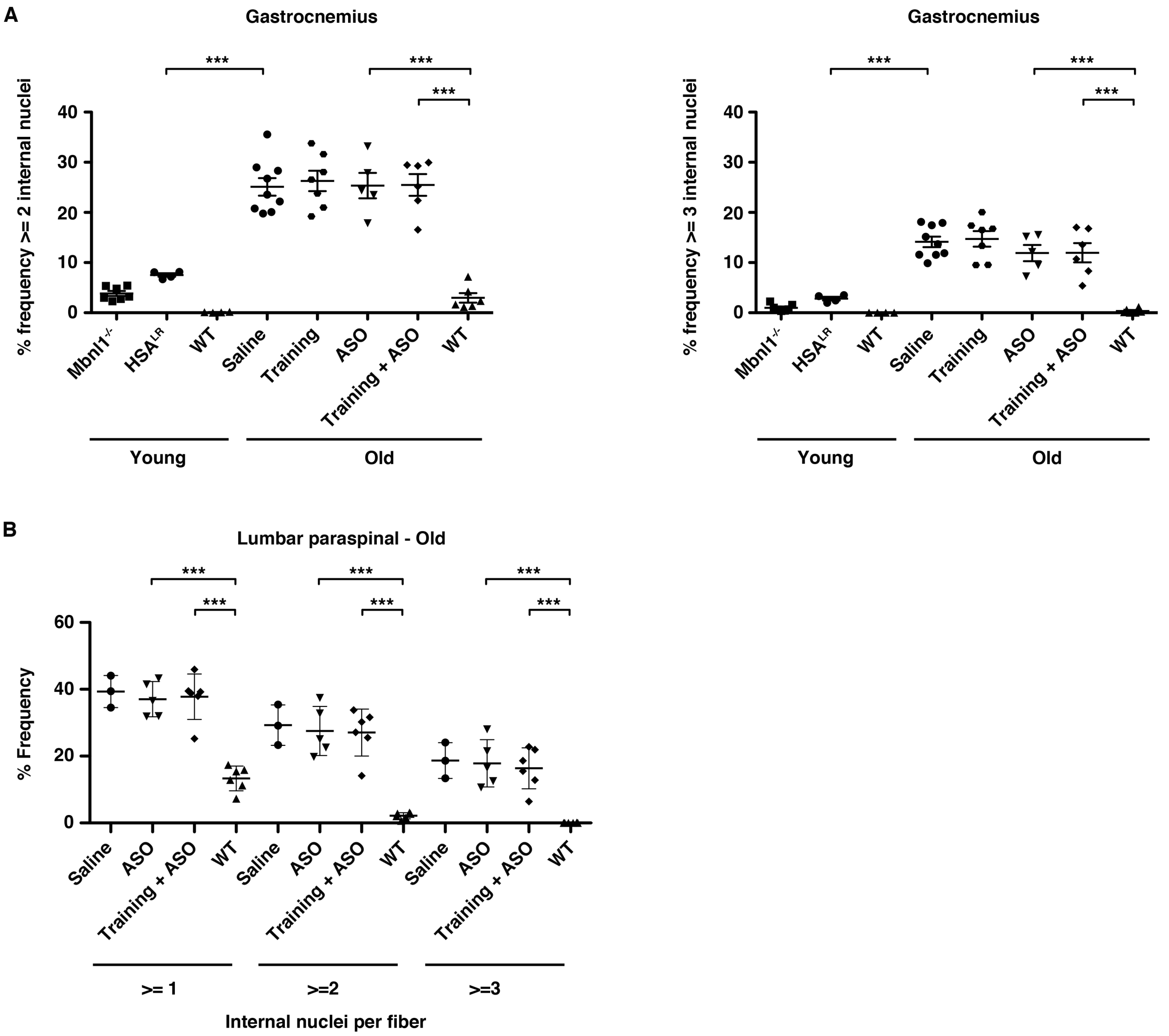

Figure S3. Relationship of fatigue to muscle histopathology. We treated old HSA^{LR} mice (~ 14 months of age) with saline (N = 9), a moderate intensity exercise training program (N = 7; Training; see Materials and Methods), ASO 445236 (N = 5), or a combination of training and ASO 445236 (N = 6) for 3 ½ months and analyzed at ~ 18 months of age. Old wild type (WT; ~ 18 months of age) (N = 6) served as controls. Muscle fibers were outlined with Alexa 647-wheat germ agglutinin (WGA) and nuclei highlighted with DAPI (see Fig 4). (A) Quantification of the percentage of gastrocnemius muscle fibers in each group that contain at least two internal nuclei (left) and at least three internal nuclei (right). Young (2 - 4 months of age) Mbnl1$^{-/-}$ (N = 6) and HSA^{LR} mice (N = 8) served as controls. *** $P < 0.001$; one-way ANOVA/Tukey. (B) Quantification of the percentage of lumbar paraspinal muscle fibers in old HSA^{LR} mice that contain at least one internal nucleus, at least two, or at least three internal nuclei. *** $P < 0.001$; one-way ANOVA/Tukey. Error bars indicate mean ± SEM. See also Figure 4.

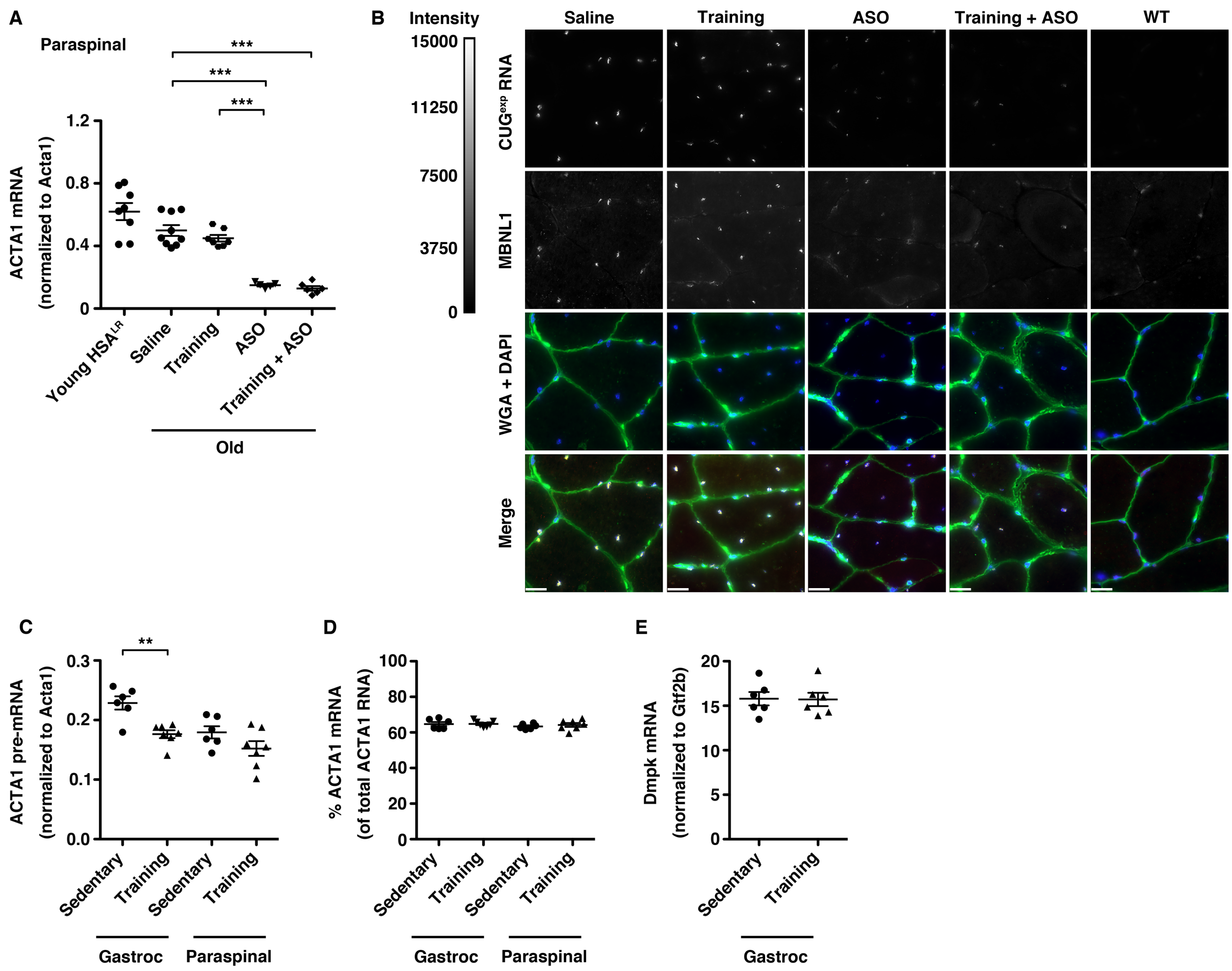

Figure S4. Molecular phenotype in muscles of DM1 mice with fatigue. We treated old HSA^{LR} mice (~ 14 months of age) with saline (N = 9), moderate intensity exercise training program (N = 7; Training; see Materials and Methods), ASO 445236 (N = 5), or a combination of training and ASO 445236 (N = 6) for 3 ½ months and analyzed at ~ 18 months of age. Young HSA^{LR} mice (2 - 4 months of age) (N = 8) served as controls. (A) Droplet digital PCR (ddPCR) quantification of *ACTA1*-CUG^{exp} transcripts in lumbar paraspinal muscles normalized to endogenous mouse *Acta1* transcripts. *** $P < 0.0001$; one-way ANOVA/Tukey. (B) Localization of CUG^{exp} RNA by fluorescence *in situ* hybridization (top row) and MBNL1 protein by immunofluorescence analysis (2nd row) in quadriceps muscles of experimental old HSA^{LR} mice and age-matched wild-type (WT) controls. Fluorescence intensity range is 0 - 15,000 grayscale units. Muscle fibers are highlighted by FITC-labeled wheat germ agglutinin (WGA; green) and nuclei by DAPI (blue). Bars = 20 μm. (C) To examine transcription of the *ACTA1* transgene, we used ddPCR to quantify *ACTA1* pre-mRNA normalized to mouse *Acta1* in gastrocnemius (gastroc) and lumbar paraspinal muscles of old mice that were sedentary (N = 6) or treated with an exercise training program (N = 7). ** $P < 0.001$; one-way ANOVA/Tukey. (D) ddPCR quantification of the percent of total *ACTA1* RNA that is mRNA in gastrocnemius and paraspinal muscles using primer probe sets specific for mRNA and pre-mRNA. The similarity of values suggests that the reduction of pre-mRNA in (B) and mRNA (Figure 5A) in mice that received exercise training is due reduced transcription of the *ACTA1*-CTG^{exp} transgene rather than enhanced metabolism of the CUG^{exp} mRNA in nuclear inclusions. (D) ddPCR quantification of *Dmpk* mRNA normalized to general transcription factor 2b (*Gtf2b*) in gastrocnemius muscle of old HSA^{LR} mice that were sedentary or treated with an exercise training regimen. Error bars indicate mean ± SEM. See also Figure 5.

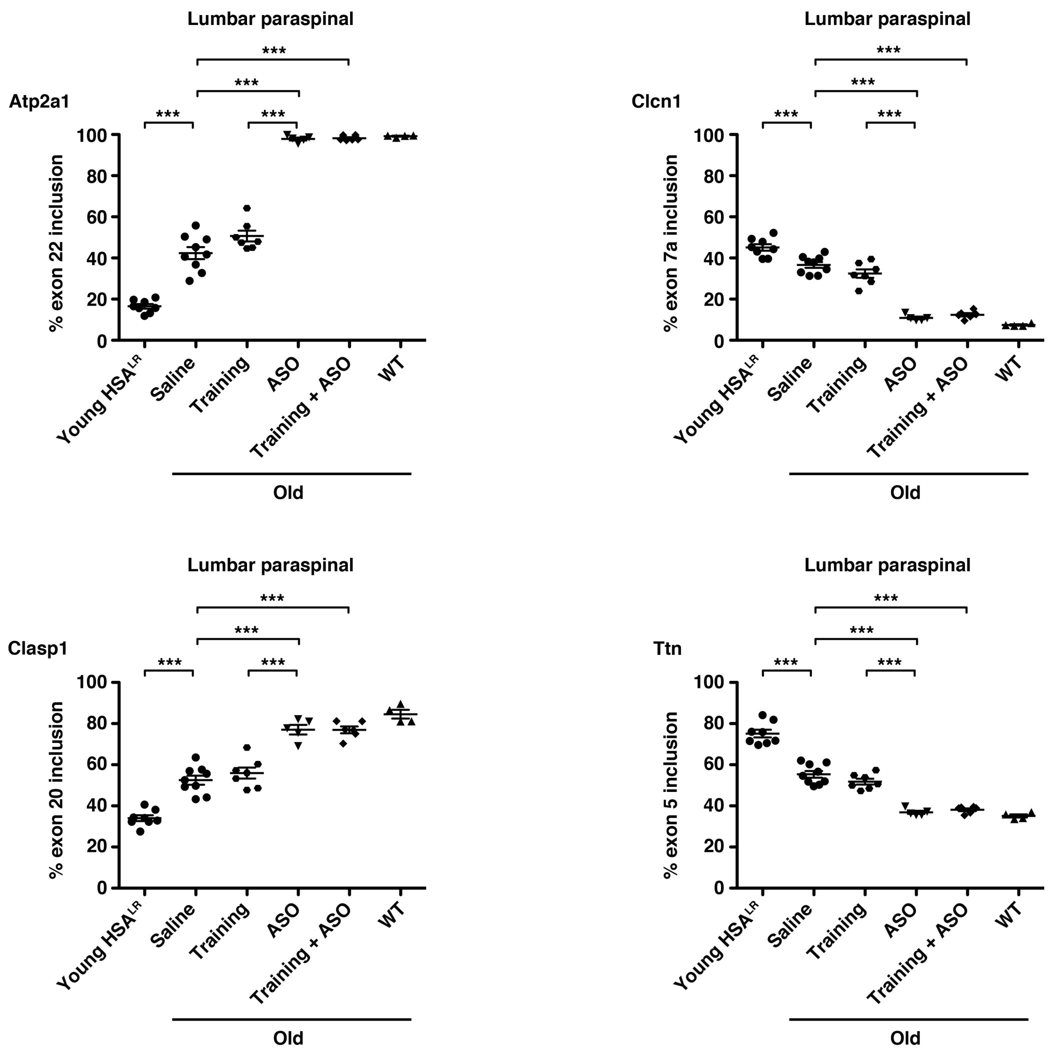

Figure S5. Alternative splicing in lumbar paraspinal muscles of DM1 mice with fatigue. We used RT-PCR and agarose gel electrophoresis to quantify alternative splicing of transcripts *Atp2a1* (upper left), *Clcn1* (upper right), *Clasp1* (lower left), and *Ttn* (lower right) in lumbar paraspinal muscles of each group. Old wild type (WT; ~ 18 months of age) (N = 4) served as additional controls. *** $P < 0.001$; one-way ANOVA/Tukey. Error bars indicate mean ± SEM. See also Figure 5.

CCACCGCAGCGGACAGCGCCAAGTGAAGCCTCGCTTCCCCTCCGCGGCGACCAGGGCCCGAGCCGAGAGTAGCAGTTGTAGCTACC
CGCCCAGgtagggcaggagttgggaggggacaggggacagggcactaccgaggggaacctgaaggactccggggcagaacccagt
cggttcacctggtcagccccaggcctgcgccctgagcgctgtgcctcgtctccggagccacacgcgctttaaaaaggaggcaagac
agtcagcctctggaaattagacttctccaaatttttctctagcccctttgggctcctttacctggcatgtaggatgtgcctaggga
gataaacggttttgctttagttgtcgccaaggcagttcccttccaaactagcgctagagcgaatgagcgagcagccaggaccaccc
attctgggtttccaacaggcgaaaaggccctttctgagtttgaaatgtcacagggttcctaacaggccactcttccctggatgggg
tgccaacgcctttcccatgggcatctccttccaccctcacgctggcccagcaagcaggcagtgctgaggccttatctccctaggtg
acagatgtggtcagggaggcgcagagaggatgggcactagcgtccagctcctggaacaggtgtcaggcagggagggcagacaggtc
ttggggaacatgttcccctggctatgtggacagaggacttctcagtggggtctcgcgaccctgtgccccttttcctggttcagggc
agcccttagccggggcaaaggtcgagaagagaacccctggtcgccgccctggcagaatttgagtggctccggcaggagatgtccct
aggttcctggggagggaggacgtcggggccagccaggcttacccccccctgccgctgagacttctgcgctgatgcaccgcgcctct
tcgcggtctccctgtccttgcagAAACTAGACACAATGTGCGACGAAGACGAGACCACCGCCCTCGTGTGCGACAATGGCTCCGGC
CTGGTGAAAGCCGGCTTCGCCGGGGATGACGCCCCTAGGGCCGTGTTCCCGTCCATCGTGGGCCGCCCCCGACACCAGgtcaggct
gcccctccgcagagggagccggctcggggtccccgcgtaagccagcctggtgccacccggagcggcgttaacgggtgcgtggtgtc
tcggctctgcagGGCGTCATGGTCGGTATGGGTCAGAAAGATTCCTACGTGGGCGACGAGGCTCAGAGCAAGAGAGGTATCCTGAC
CCTGAAGTACCCTATCGAGCACGGCATCATCACCAACTGGGATGACATGGAGAAGATCTGGCACCACACCTTCTACAACGAGCTTC
GCGTGGCTCCCGAGGAGCACCCCACCCTGCTCACCGAGGCCCCCCTCAATCCCAAGGCCAACCGCGAGAAGATGACCCAGATCATG
TTTGAGACCTTCAACGTGCCCGCCATGTACGTGGCCATCCAGGCCGTGCTGTCCCTCTACGCCTCCGGCAGGACCACCGgtgagtg
cccgctgcccccagtcccctctcccgcccccgcccccgcccccgcccccgccgctagcgctgagagcctagcctcggcctcgcccc
cagccactcactctctcccgcgcgcgcacagGCATCGTGCTGGACTCCGGCGACGGCGTCACCCACAACGTGCCCATTTATGAGGG
CTACGCGCTGCCGCACGCCATCATGCGCCTGGACCTGGCGGGCCGCGATCTCACCGACTACCTGATGAAGATCCTCACTGAGCGTG
GCTACTCCTTCGTGACCACAGgtgcgcggcgcccctgcaccccgggcgggagggccggcggcggcccctgagtgagggctcctctc
ctgcttctgccctccgcagCTGAGCGCGAGATCGTGCGCGACATCAAGGAGAAGCTGTGCTACGTGGCCCTGGACTTCGAGAACGA
GATGGCGACGGCCGCCTCCTCCTCCTCCCTGGAAAAGAGCTACGAGCTGCCAGACGGGCAGGTCATCACCATCGGCAACGAGCGCT
TCCGCTGCCCGGAGACGCTCTTCCAGCCCTCCTTCATCGgtgagccccgctcgccctcgccccggcccccaggccccgccggcccc
cggcgccccgagcttctgctcacgctccccgccgcggtccccagGTATGGAGTCGGCGGGCATTCACGAGACCACCTACAACAGCA
TCATGAAGTGTGACATCGACATCAGGAAGGACCTGTATGCCAACAACGTCATGTCGGGGGGCACCACGATGTACCCTGGGATCGCT
GACCGCATGCAGAAAGAGATCACCGCGCTGGCACCCAGCACCATGAAGATCAAGgtgggtggtggcctgcgcgggctgtcggcggg
gtgggctccagggtgaggtctccccacctcacgcgctgtcttgcagATCATCGCCCCGCCGGAGCGCAAATACTCGGTGTGGATCG
GCGGCTCCATCCTGGCCTCGCTGTCCACCTTCCAGCAGATGTGGATCACCAAGCAGGAGTACGACGAGGCCGGCCCTTCCATCGTC
CACCGCAAATGCTTCTAGACACACTCCACCTCCAGCACGCGACTTCTCAGGACGACGAATCTTCTCAATGGGGGGGCGGCTGAGCT
CCAGCCACCCCGCAGTCACTTTCTTTGTAACAACTTCCGT**TGCTGCCATCGTAAACTGAC**ACAGTGTTTATAACGTCTGCTGCTGC
TGCTGCTGCTGCTGCTGCTGCTGCTGCTGCTGCTGCTGCTGCTGCTGCTGCTGCTGCTGCTGCTGCTGCTGCTGCTGCTGCTG
CTGCTGCTGCTGCTGCTGCTGCTGCTGCTGCTGCTGCTGCTGCTGCTGCTGCTGCTGCTGCTGCTGCTGCTGCTGCTGCTGCT
GCTGCTGCTGCTGCTGCTGCTGCTGCTGCTGCTGCTGCTGCTGCTGCTGCTGCTGCTGCTGCTGCTGCTGCTGCTGCTGCTGC
TGCTGCTGCTGCTGCTGCTGCTGCTGCTGCTGCTGCTGCTGCTGCTGCTGCTGCTGCTGCTGCTGCTGCTGCTGCTGCTGCTG
CTGCTGCTGCTGCTGCTGCTGCTGCTGCTGCTGCTGCTGCTGCTGCTGCTGCTGCTGCTGCTGCTGCTGCTGCTGCTGCTGCT
GCTGCTGCTGCTGCTGCTGCTGCTGCTGCTGCTGCTGCTGCTGCTGCTGCTGCTGCTGCTGCTGCTGCTGCTGCTGCTGCTGC
TGCTGCTGCTGCTGCTGCTGCTGCTGCTGCTGCTGCTGCTGCTGCTGCTGCTGCTGCTGCTGCTGCTGCTGCTGCTGCTGCTG
CTGCTGCTGCTGCTGCTGCTGCTGCTGCTGCTGCTGCTGCTGCTGCTGCTGCTGCTGCTGCTGCTGCTGCTGCTGCTGCTGCT
GCTGCTGCTGCTGCTGCTGCTGCTGCTGCTGCTGCTGCTGCTGCTGCTGGTACATACATTAACTTATTACCTCATTTTGTTAT
TTTTC**GAAACAAAGCCCTGTGGAAG**AAAATGGAAAACTTGAAGAAGCATTAAAGTCATTCTGTTAAGCTGCGT

Figure S6. *ACTA1* genomic sequence (UCSC genome browser) with 185 CTG repeats inserted into *Bsr* GI restriction site in the 3′ UTR that together comprise the transgene in the HSA^{LR} mouse model (see main text reference 29). Exon sequence is shown in upper case, intron sequence in lower case. To determine the CTG repeat length, we used a left primer that targets sequence 36 base pairs (bp) upstream of the CTG repeat sequence (turquiose background) and a right primer that targets sequence 59 bp down-stream of the CTG repeat sequence (yellow background). Therefore, the total sequence amplified outside of the CTG repeat is 36 bp + 59 bp = 95 bp. Based on an RT-PCR product size of approximately 650 bp evident in DNA from tail biopsy and gastrocnemius DNA (see Figure 7), the CTG repeat (underlined) in this model is approximately (650 bp - 95 bp non-CTG repeat sequence) = 555 bp. If the CTG repeat sequence = 555 bp, then the number of CTG repeats = 555/3 = approximately 185.

Table S1 ddPCR primer probe sets (PP sets). Probes for *ACTA1* mRNA and pre-mRNA, and mouse *Dmpk* mRNA were 5′-labeled with Fam, and 3′-labeled with Black Hole Quencher-1 (BHQ-1) (Biosearch Technologies). The amplicon size in base pairs (bp) is shown for each. We purchased an assay for mouse *Acta1* that uses a probe 5′-labeled with Hex and produces an amplicon of 117 bp (Bio-Rad; unique assay ID: dMmuCPE5088325).

PP set	Left primer (5′ - 3′)	Right primer (5′ - 3′)	Probe (5′ - 3′)	Amplicon size (bp)
ACTA1 mRNA	GTAGCTACCCGCCCAGAAACT	CCAGGCCGGAGCCATT	ACCACCGCCCTCGTGTGCG	83
ACTA1 pre-mRNA	ctgtccttgcagAAACTAGACAC	CCAGGCCGGAGCCATT	ACCACCGCCCTCGTGTGCG	79
mouse Dmpk mRNA	GACATATGCCAAGATTGTGCACTAC	CACGAATGAGGTCCTGAGCTT	AACACTTGTCGCTGCCGCTGGC	92

Table S2. PCR primers used to measure alternative splicing or estimate *ACTA1*-CTG^{exp} repeat length. PCR product size in base pairs (bp) with exon inclusion (+ ex) and exon exclusion (- ex) is shown.

Gene	Left primer (5′ - 3′)	Right primer (5′ - 3′)	Target exon(s)	+ ex size (bp)	- ex size (bp)
Atp2a1	GCTCATGGTCCTCAAGATCTCAC	GGGTCAGTGCCTCAGCTTTG	22	218	176
Clasp1	GTCGACGACAGGATCTCTCC	GAGCTCTGCCGTCTCGTG	20	198	174
Clcn1	TGAAGGAATACCTCACACTCAAGG	CACGGAACACAAAGGCACTG	7a	424	345
Titin	GTGTGAGTCGCTCCAGAAACG	CCACCACAGGACCATGTTATTTC	5	556	253
ACTA1-CTG	TGCTGCCATCGTAAACTGAC	CTTCCACAGGGCTTTGTTTC	CTG expansion	n/a	n/a

Movie S1. Monitoring spontaneous mouse movements in the x-, y-, and z-planes using an acrylic cage and infrared lasers. We tested all mice at night or synchronized to a reverse light cycle (shifted 12-hour:12-hour dark:light) room so that at the time of testing, the mice were behaviorally most active (see Materials and Methods). Representative monitoring is shown under red light.

Movie S2. Moderate intensity treadmill walking exercise. We exercised mice using a treadmill on a flat surface (no incline) at 11.5 m/min for 30 minutes, six days per week for 15 weeks (see Materials and Methods). Representative exercise is shown.
