# Supplementary material for: Antisense oligonucleotide and adjuvant exercise therapy reverse fatigue in old mice with myotonic dystrophy
Source: Mol Ther Nucleic Acids. 2020 Nov 26;23:393–405. doi: 10.1016/j.omtn.2020.11.014 (PMC7787993; doi:10.1016/j.omtn.2020.11.014)
Supplement: Document S2. Article plus supplemental information [file mmc4.pdf]

Molecular Therapy
Nucleic Acids
Original Article

Antisense oligonucleotide and adjuvant exercise therapy reverse fatigue in old mice with myotonic dystrophy

Ningyan Hu,[1] Eunjoo Kim,[1] Layal Antoury,[1] Jia Li,[2] Paloma González-Pérez,[1] Seward B. Rutkove,[2] and Thurman M. Wheeler[1]

[1]Department of Neurology, Massachusetts General Hospital and Harvard Medical School, Boston, MA 02129, USA; [2]Beth Israel Deaconess Medical Center and Harvard Medical School, Boston, MA 02215, USA

Patients with myotonic dystrophy type 1 (DM1) identify chronic fatigue as the most debilitating symptom, which manifests in part as prolonged recovery after exercise. Clinical features of DM1 result from pathogenic gain-of-function activity of transcripts containing an expanded microsatellite CUG repeat (CUG^{exp}). In DM1 mice, therapies targeting the CUG^{exp} transcripts correct the molecular phenotype, reverse myotonia, and improve muscle pathology. However, the effect of targeted molecular therapies on fatigue in DM1 is unknown. Here, we use two mouse models of DM1, age-matched wild-type controls, an exercise-activity assay, electrical impedance myography, and therapeutic antisense oligonucleotides (ASOs) to show that exaggerated exercise-induced fatigue progresses with age, is unrelated to muscle fiber size, and persists despite correction of the molecular phenotype for 3 months. In old DM1 mice, ASO treatment combined with an exercise training regimen consisting of treadmill walking 30 min per day 6 days per week for 3 months reverse all measures of fatigue. Exercise training without ASO therapy improves some measures of fatigue without correction of the molecular pathology. Our results highlight a key limitation of ASO monotherapy for this clinically important feature and support the development of moderate-intensity exercise as an adjuvant for targeted molecular therapies of DM1.

INTRODUCTION

Myotonic dystrophy (dystrophia myotonica [DM]) is the most common muscular dystrophy in adults, with a worldwide prevalence of between 1:3,000 and 1:8,000.[1,2] Characteristics of this multisystem disorder include myotonia (delayed muscle relaxation due to repetitive action potentials), chronic fatigue, progressive weakness, muscle wasting, cardiac conduction disturbance, insulin resistance, neuropsychiatric symptoms, and gonadal atrophy. Inheritance is autosomal dominant. DM type 1 (DM1) results from a CTG repeat expansion in the 3′ untranslated region of the *DM protein kinase* (*DMPK*) gene on chromosome 19q.[3] DM1 disease severity and rate of progression correlate with allele length of the CTG repeat.[4,5] Clinical manifestations of DM1 result from accumulation of *DMPK*-CUG^{exp} transcripts in skeletal muscle and other affected tissues. Thus far, no treatment that alters the disease course in patients has been identified, although targeted molecular therapies that neutralize the pathogenic CUG^{exp} transcripts have shown promise in DM1 mouse models.[6–12]

Fatigue affects more than 90% of DM1 patients and is the symptom that has the greatest overall impact on their lives, even surpassing limitations of mobility.[13] In general terms, fatigue is a state of depressed responsiveness resulting from protracted activity and requiring an appreciable recovery time.[14] Clinically, it manifests as difficulty initiating or maintaining voluntary activities and is distinct from muscle weakness or depressed mood.[15] Fatigue can be divided into central and peripheral components. The main features of central fatigue are enhanced perception of effort and a limited ability to sustain mental and physical activities.[15] Peripheral fatigue is a reduction in the force of muscle fiber contraction and follows repeated contraction or direct stimulation of the muscle.[14] Previous studies suggest that fatigue in DM1 is more central than peripheral,[15–21] is separate from daytime sleepiness,[22] and includes symptoms such as decreased energy, a sensation of tired muscles, and prolonged recovery after exercise.[13]

More than 90% of patients with a second type of muscular dystrophy, facioscapulohumeral muscular dystrophy (FSHD), also experience fatigue.[23] A recent study in FSHD patients found that either aerobic exercise training or cognitive behavioral therapy can reduce chronic fatigue.[24] Exercise training also has been evaluated as a non-pharmacological therapy for chronic fatigue in patients with chronic fatigue syndrome/myalgic encephalomyelitis[25] and multiple sclerosis,[26] and in cancer survivors.[27] The effect of exercise therapy on fatigue in DM1 patients is unknown.[28] In this study, we examined the effect of a 3-month training program that consisted of treadmill walking

Received 24 July 2020; accepted 17 November 2020;
https://doi.org/10.1016/j.omtn.2020.11.014.

Correspondence: Thurman M. Wheeler, Department of Neurology, Massachusetts General Hospital and Harvard Medical School, CNY 149 – 6323, 149 13th St., Boston, MA 02129, USA.
E-mail: twheeler1@mgh.harvard.edu

© 2020 The Author(s).
This is an open access article under the CC BY license (http://creativecommons.org/licenses/by/4.0/).

combined with a therapeutic antisense oligonucleotide (ASO) on post-exercise fatigue in DM1 transgenic mice.

RESULTS

Exaggerated fatigue in two DM1 mouse models

The human skeletal actin long repeat (HSA^{LR}) transgenic mouse model of DM1 was designed to test the hypothesis that expanded CUG repeat-bearing RNA is toxic for muscle cells.[29] This model contains an expanded CTG repeat in the 3′ untranslated region of the human skeletal actin (*ACTA1*) gene. The *ACTA1* transgene restricts expression of CUG^{exp} transcripts to skeletal muscle tissue, which accumulate in nuclear inclusions that resemble those found in skeletal muscle of individuals with DM1. Similar to DM1 patients, the CUG^{exp} RNA in these mice initiates pathology by sequestering RNA binding proteins in the muscleblind-like (MBNL) family, resulting in mis-regulation of alternative splicing of several dozen transcripts. The pathogenic activity of CUG^{exp} RNA leads to myotonia, histopathologic signs of muscular dystrophy, and splicing patterns that are similar to human DM1. Support for this RNA-dominant disease process comes from observations that *Mbnl1* knockout (*Mbnl1*$^{-/-}$) mice[30] display multi-systemic features that resemble DM1 and that defects of alternative splicing induced by expression of CUG^{exp} RNAs or by ablation of the *Mbnl1* gene are very similar.[31,32]

In the HSA^{LR} model, the restriction of CUG^{exp} RNA to skeletal muscle permits the study of the aspect of fatigue in DM1 that is due to intrinsic muscle pathology. To test whether HSA^{LR} mice develop fatigue, we used an exercise-activity assay. First, we placed 2- to 3-month-old HSA^{LR}, *Mbnl1*$^{-/-}$, and wild-type (WT) control mice on a treadmill for 10 min up a 15-degree incline at a speed of 20 m per min. Immediately post-exercise, we monitored mouse movement in the x, y, and z planes for 30 min using an acrylic cage and infrared lasers (see Video S1). Quantitative activity measurements were collected in 1-min intervals and included the number of single-rearing events with 1 s between each event (vertical breaks), total counts from the z-plane sensor (vertical counts), total distance traveled (cm), ambulatory time in seconds (s), rest time (s), and the total time spent on small rapid movements such as scratching, grooming, or other stereotypic non-ambulatory movements (stereotypic time; s) during the 30-min (1,800 s) monitoring period. In HSA^{LR} mice, post-exercise fatigue was mild, with small deficits in vertical breaks, ambulatory time, and distance traveled relative to age-matched WT controls (Figure 1A). In *Mbnl1*$^{-/-}$ mice, post-exercise spontaneous activity was significantly lower than in age-matched HSA^{LR} mice and less than half of that in WT (Figure 1A). Stereotypic time was similar in all groups (Figure S1).

To examine the relationship between exaggerated fatigue in DM1 mice and muscle structural abnormalities, we used electrical impedance myography (EIM) to estimate myofiber size non-invasively. EIM measures the flow of current through muscle tissue, focusing on rate, speed, efficiency, frequency, and direction.[34] Prior EIM studies have found that the center frequency (f_c), an index that is inversely related to cell size, and the 50-kHz electric phase angle, which refers to the time shift of electric current that occurs as a result of its passage through tissue at a frequency of 50 kHz, are sensitive measures of muscle fiber size in models of atrophy and hypertrophy.[35,36] We found that the f_c was significantly decreased and the 50-kHz electric phase angle significantly increased in gastrocnemius muscles of young HSA^{LR} mice as compared to *Mbnl1*$^{-/-}$ mice or age-matched WT controls (Figure 1B), both features consistent with increased myofiber size. To evaluate whether EIM parameters were accurate indicators of myofiber size in these models, we measured minimum Feret's diameter[33] in muscle tissue sections. Fiber diameter distribution appeared similar in *Mbnl1*$^{-/-}$ and WT but shifted toward hypertrophy in young HSA^{LR} mice, with mean values approximately 25% larger than in the other two groups (Figures 1C–1E).

Fatigue in DM1 transgenic mice progresses with age

In humans with DM1, symptoms of fatigue worsen with age.[13] To explore whether fatigue progresses in the HSA^{LR} model, we tested fatigue in aged mice. In the absence of preceding exercise, spontaneous activity in 15-month-old HSA^{LR} mice appeared similar to age-matched WT controls (Figure 2A). Post-exercise, old HSA^{LR} mice demonstrated exaggerated fatigue by several measures as compared to age-matched WT controls, including less frequent vertical activity, shorter distance traveled, and longer resting time (Figure 2A). Comparison of the pre- and post-exercise data for each individual mouse found that post-exercise spontaneous activity in HSA^{LR} mice was less than half that in WT for all measurements of fatigue (Figure 2B). Post-exercise stereotypic time appeared similar in both groups (Figure S1). We were unable to test fatigue in old *Mbnl1*$^{-/-}$ mice because, in our experience, these mice tend to die without warning sometime between 2 and 6 months of age. To determine whether fatigue progressed with age, we performed head-to-head comparisons of post-exercise activity in young and old mice. The differences in measurements of vertical breaks, ambulatory time, and rest time between old HSA^{LR} versus old WT were significantly greater (30%–50% change) than the statistically non-significant differences between young HSA^{LR} versus young WT. Spontaneous activity also was significantly lower (30%–60% change) in old HSA^{LR} versus young HSA^{LR}, while differences in these measures between young WT versus old WT were statistically non-significant (Figure 2C).

Treatment effect on fatigue

If exaggerated fatigue in aged HSA^{LR} mice is due to pathogenic activity of CUG^{exp} transcripts in muscle tissue, then correction of the underlying molecular derangements may reduce fatigue. To date, ASOs are the most promising therapeutic agents for DM1.[6–8,11,37] We treated 14-month-old HSA^{LR} mice for 3 months with saline or a therapeutic ASO that targets the *ACTA1*-CUG^{exp} transcripts for cleavage via the RNase H pathway and reverses the molecular phenotype in young HSA^{LR} mice.[6,8] Surprisingly, all measures of fatigue appeared similar in the saline- and ASO-treated groups (Figure 3A). In a third group, a moderate-intensity exercise training regimen that consisted of treadmill walking at a speed of 11.5 m/min, zero degree incline for

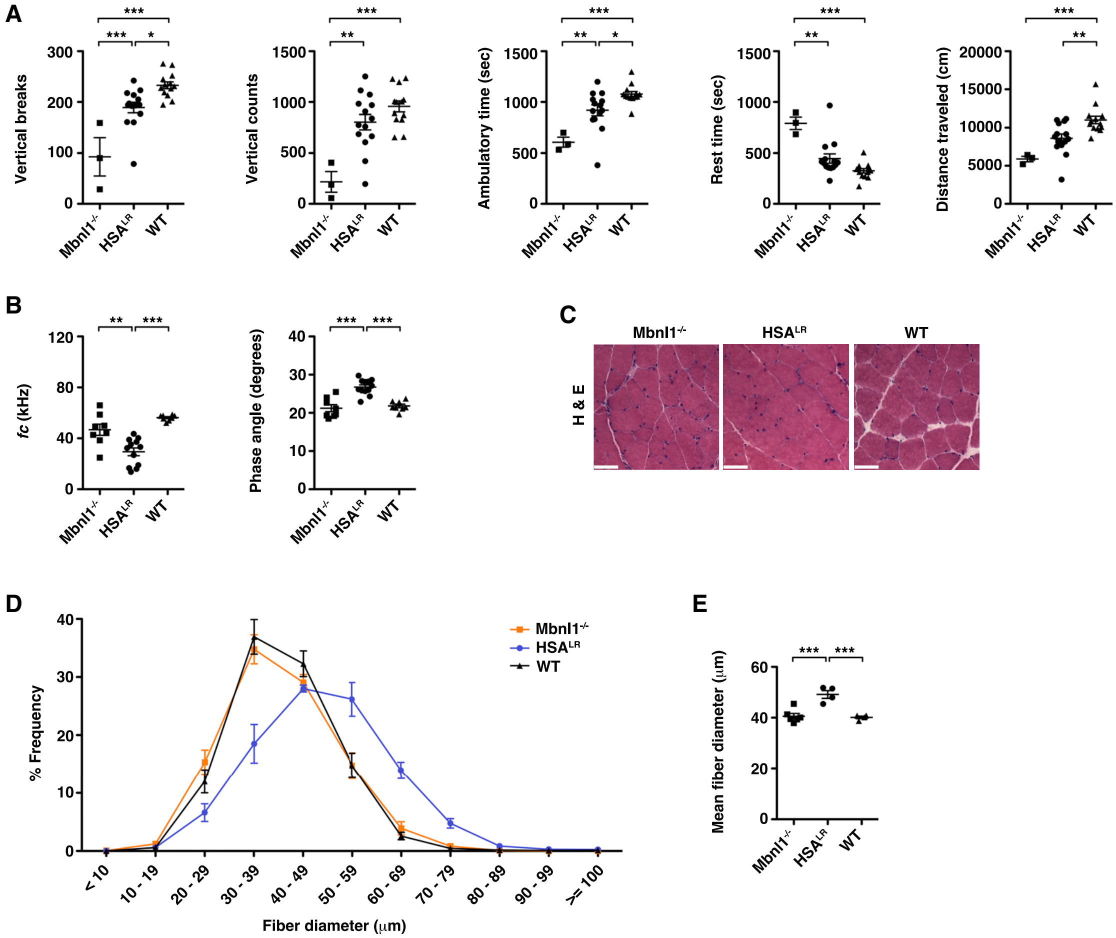

Figure 1. Muscle fatigue in two mouse models of DM1 is independent of muscle fiber size

(A) We examined spontaneous x-, y-, and z-plane activity of untreated 2- to 3-month-old *Mbnl1* knockout (–/–; N = 3), HSA^{LR} (N = 14), and WT (N = 13) mice for 30 min immediately following treadmill exercise for 10 min uphill at a 15-degree angle (see Materials and Methods). The number of single-rearing events with 1 s between each event (vertical breaks), total counts from the z sensor (vertical counts), ambulatory time (s), rest time (s), and total distance traveled (cm) during the 30-min (1,800 s) monitoring period are shown. Individual data points represent the mean of three trials performed on separate days of each individual mouse. ***p < 0.001; **p < 0.01; *p < 0.05; one-way ANOVA/Tukey. (B) Non-invasive EIM measurements of gastrocnemius muscles under general anesthesia as estimates of muscle fiber size and structural pathology. f_c (left) and phase angle (right) with current applied in a transverse direction with respect to muscle fiber orientation. ***p < 0.001; **p < 0.01; one-way ANOVA/Tukey. (C) Representative hematoxylin and eosin (H&E) staining of transverse gastrocnemius muscle cryosections. Bars, 50 μm. (D) Minimum Feret's muscle fiber diameter, defined as the minimum distance between parallel tangents,[33] in 8-μm cryosections of gastrocnemius muscles in each group shown in (B) and (C). (E) Mean fiber diameter in each group using the data in (D). ***p < 0.001; one-way ANOVA/Tukey. Error bars indicate mean ± SEM.

30 min per day 6 days per week for 15 weeks[38] (see Video S2) improved fatigue resistance in five of the seven mice, although results for the entire group fell short of statistical significance (Figure 3A). Mice treated with ASO combined with the exercise-training regimen were the only group that showed a consistent improvement in all measures of fatigue (Figure 3A). The EIM parameter f_c appeared similar regardless of treatment and was significantly reduced in all groups as compared to age-matched WT controls, while electric phase angle measurements in HSA^{LR} mice appeared similar regardless of treatment (Figure 3B). Histologic measurement of muscle fiber diameter showed a size distribution in the training, ASO alone, and training combined with ASO treatment groups that is intermediate between that in the saline-treated group and WT controls (Figures 3C–3E; Figure S2).

The age-related progression of fatigue and its differential response in the treatment groups suggested the possibility that intrinsic structural abnormalities of skeletal muscle also differed between groups. We found that capillary density was 38% greater in saline-treated old HSA^{LR} mice than in young HSA^{LR} or *Mbnl1*$^{-/-}$ mice and 78% greater than in age-matched WT (Figures 4A and 4B). Capillary density also showed a non-significant reduction in the training group and a 20% reduction in old mice treated with either ASO or ASO combined with exercise training (Figures 4A and 4B). The percentage of fibers that

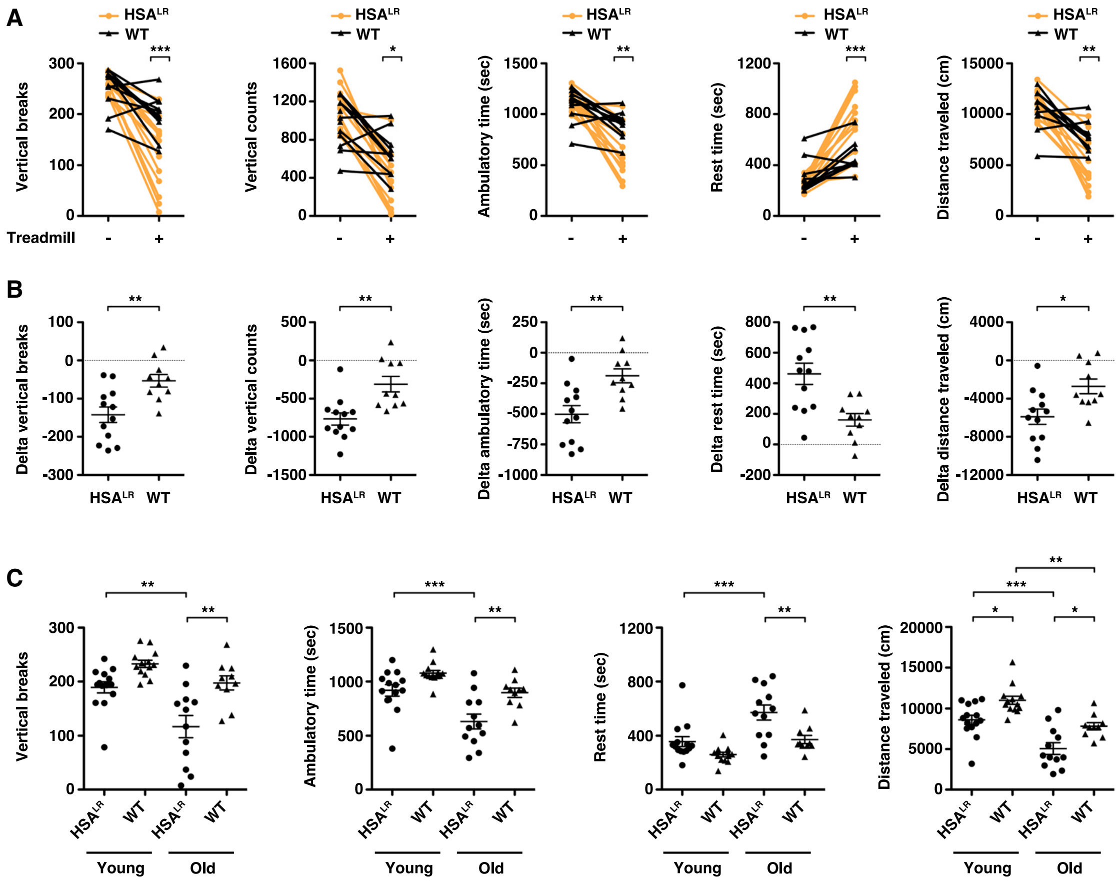

Figure 2. Muscle fatigue as a progressive phenotype in DM1 transgenic mice

We examined spontaneous x-, y-, and z-plane activity of untreated 15-month-old HSA^{LR} (N = 12; orange circles) and WT (N = 10; black triangles) mice for 30 min without preceding treadmill exercise (–) or immediately following treadmill exercise (+) for 10 min uphill at a 15-degree angle (see Materials and Methods). (A) Quantification of single-rearing events with 1 s between each event (vertical breaks), total counts from the z sensor (vertical counts), ambulatory time (s), rest time (s), and total distance traveled (cm) during the 30-min (1,800 s) monitoring period. Individual data points represent the mean of three trials performed on separate days of each individual mouse. ***p < 0.001, **p < 0.01, *p < 0.05, all HSA^{LR} versus WT post-exercise; two-way ANOVA. (B) The difference (delta) between the pre- and post-exercise measurements of each individual mouse shown in (A). **p = 0.003, delta vertical breaks and delta ambulatory time; and 0.002, delta vertical counts and delta rest time; *p = 0.011, delta distance traveled; two-tailed t tests. (C) Post-exercise spontaneous activity measurements in 2- to 3-month-old (young) HSA^{LR} and WT and 15-month-old (old) HSA^{LR} and WT mice. ***p < 0.001, p < 0.01, and *p < 0.05; one-way ANOVA/Tukey. Note that the differences between young HSA^{LR} and young WT and between young WT and old WT were non-significant for vertical breaks, ambulatory time, and rest time. Error bars indicate mean ± SEM.

contain internal nuclei was more than double in saline-treated old HSA^{LR} mice as compared to saline-treated young HSA^{LR} or *Mbnl1*$^{-/-}$ mice and was unchanged regardless of treatment (Figure 4C; Figure S3).

Molecular phenotype in mice with fatigue

The persistence of fatigue and myopathy despite drug treatment suggested the possibility that ASOs may be ineffective in old HSA^{LR} mice. To examine ASO drug target engagement, we quantified *ACTA1*-CUG^{exp} transcripts by droplet digital PCR (ddPCR).[11] *ACTA1*-CUG^{exp} transcript abundance appeared similar in muscles of old mice treated with ASO alone or ASO combined with an exercise-training regimen (Figure 5A; Figure S4). Unexpectedly, we found that baseline *ACTA1*-CUG^{exp} RNA levels were lower in saline-treated old HSA^{LR} mice than in young HSA^{LR} mice (Figure 5A; Figure S4). Muscles of old mice in the exercise-training group tended to have a lower abundance of *ACTA1*-CUG^{exp} transcripts than in saline-treated old mice, although the difference was statistically non-significant. Ribonuclear foci of CUG^{exp} RNA and MBNL1 protein appeared similar by quantitative imaging of muscle-tissue sections in groups treated with saline or exercise training and were less intense in groups treated with ASO or ASO + training (Figure S4). We next investigated whether the small decrease of *ACTA1* transcript abundance in muscles of the exercise-alone group was due to reduced transcription of the *ACTA1* transgene or enhanced degradation of *ACTA1* mRNA. Using a ddPCR primer probe set targeting the *ACTA1* intron 1-exon 2 splice site, we found that pre-mRNA levels were reduced in old mice treated with exercise alone to a similar degree as the mRNA and that the

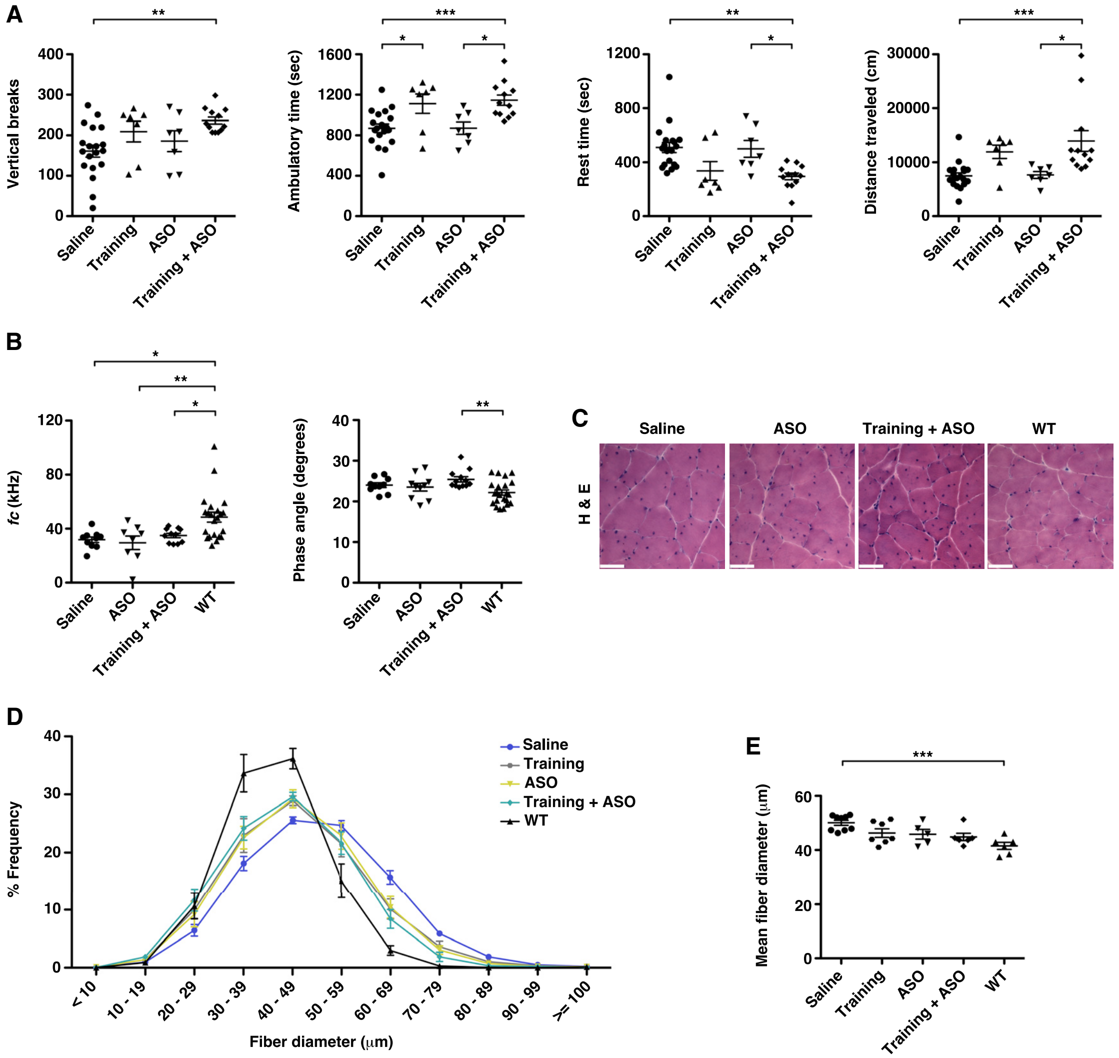

Figure 3. Effect of exercise training and targeted molecular therapy on muscle fatigue

We treated ~14 month-old HSA[LR] mice with a 15-week course of saline, a moderate-exercise training program (see Materials and Methods), a therapeutic ASO, or a combination of training + ASO and performed a post-treatment exercise-activity assay at 17.5–18 months of age. (A) Vertical breaks, ambulatory time (s), rest time (s), and total distance traveled (cm) in each group. Individual data points represent the mean of three trials performed on separate days for each individual mouse. ***$p < 0.001$, **$p = 0.01$, and *$p < 0.05$; one-way ANOVA/Tukey. (B) Non-invasive EIM measurements of gastrocnemius muscles under general anesthesia as estimates of muscle-fiber size and structural pathology. f_c (left) and phase angle (right) with current applied in a transverse direction with respect to muscle-fiber orientation. **$p < 0.01$; *$p < 0.05$; one-way ANOVA/Tukey. (C) Representative hematoxylin and eosin (H&E) staining of transverse gastrocnemius muscle cryosections. Bars, 50 μm. (D) Minimum Feret's muscle-fiber diameter, defined as the minimum distance between parallel tangents,[33] in 8-μm cryosections of gastrocnemius muscles in each group shown in (B) and (C). (E) Mean fiber diameter in each group using the data in (D). ***$p < 0.001$; one-way ANOVA/Tukey. Error bars indicate mean ± SEM.

proportion of the total *ACTA1*-CUG[exp] transcripts that were mRNA identical in all groups (Figure S4). We also explored whether exercise training may lead to differences in expression of *Dmpk*, which is the gene that carries the CUG[exp] in humans with DM1. Using ddPCR, we found that *Dmpk* transcript levels were identical in mice that were sedentary or received exercise training for 3 months (Figure S4).

Evaluation of alternative splice events enables a convenient measurement of molecular disease status in DM1 patients[39,40] or therapeutic drug activity in DM1 mice.[8,11] To examine the relationship between fatigue and molecular phenotype, we used RT-PCR to measure alternative splicing of *Atp2a1*, *Clasp1*, *Clcn1*, and *Ttn*, transcripts that are mis-regulated in DM1 patients and young HSA[LR] mice.[11,31,41–45] In saline-treated old HSA[LR] mice that feature significantly lower CUG[exp] RNA levels than in untreated young mice, exon inclusion percentage was intermediate between untreated young mice and WT (Figures 5B and 5C; Figure S5). Splicing patterns of all four transcripts in mice treated with ASO alone or ASO combined with exercise training

appeared similar to those in WT. Mice in the training group also showed mild improvement of splicing, consistent with the trend for slightly lower *ACTA1*-CUG^{exp} RNA levels.

CTG repeat length in aged muscle tissue

In human DM1, expanded CTG repeats are highly unstable, tending to increase in an age- and tissue-dependent manner, and in muscle tissue, somatic expansion of the CTG length can precede progression of weakness.[39,46–49] To test whether progression of fatigue or myopathy in HSA^{LR} mice may be explained by age-related somatic expansion of the CTG repeat, we performed small-pool PCR and Southern blotting[50] of tail biopsy DNA obtained at weaning and gastrocnemius muscle tissue DNA from the same mice at age 18 months. Using a left primer that amplifies 36 base pairs (bp) upstream of the CTG repeat insertion and a right primer that amplifies 59 bp downstream of the CTG repeat insertion, we identified a single band of approximately 650 bp that appeared identical in young tail DNA and aged gastrocnemius muscle tissue DNA (Figures 6A and 6B). The sequence amplified outside the CTG expansion is a total of 36 + 59 = 95 bp. Therefore, the CTG repeat sequence is 650 − 95 = 555 bp, which corresponds to 555/3 = 185 CTG repeats (Figure S6), 26% shorter than the 250 CTG repeats originally reported in this model.[29]

DISCUSSION

Fatigue is common in many neuromuscular disorders and is the symptom that has the greatest overall impact on the lives of DM1 patients[13]. This is the first study to identify and rescue fatigue in a model of DM1. We found that combining ASO therapy with a moderate-intensity exercise-training regimen reversed both the molecular phenotype and all measures of *in vivo* post-exercise fatigue in old HSA^{LR} mice. The failure of ASO monotherapy to improve fatigue in this model suggests a key limitation of targeted molecular therapies for the treatment of this clinically important feature. Moderate-intensity exercise training, a non-pharmacological intervention that reduces chronic fatigue in patients with FSHD,[24] also showed a trend for improvement in fatigue of old HSA^{LR} mice but without correction of the molecular or histologic phenotype. Our results support the development of moderate-intensity exercise training as both a stand-alone therapy for fatigue and, eventually, an adjuvant for future ASO and other molecular therapies in DM1 patients. Initiation of therapy in young individuals with mild manifestations likely would achieve optimal clinical benefit.

The moderate-intensity exercise regimen that we used for the mice consisted of treadmill walking on a flat surface for 30 min per day 6 days per week, for a total of 180 min per week. This is comparable to the physical activity guidelines issued by the US Department of Health and Human Services, Canadian Society for Exercise Physiology, the UK Department of Health and Social Care, and the European Union Sport Ministers that all recommend at least 150 min of moderate-intensity aerobic activity, such as brisk walking, for adults each week.[51–54] Given that most DM1 patients are ambulatory, a similar regimen should be clinically feasible.

In individuals with classical adult-onset DM1, clinical manifestations such weakness and fatigue are progressive: they are absent or unnoticeable in youth and gradually worsen with age. In this study, we demonstrate that fatigue and myopathy in the HSA^{LR} model also are progressive: they are mild in youth and gradually worsen with age. The tissue specificity and expression stage of the transgene in the HSA^{LR} model enabled determination that the cumulative life-long effects of expanded CUG repeats in muscle tissue are sufficient to cause post-exercise fatigue, independent of pathology in non-muscle tissues. The substantially greater fatigue in *Mbnl1*$^{-/-}$ mice, as compared to age-matched HSA^{LR} mice, suggests the possibility that the combined absence of MBNL1 activity in the skeletal muscle, peripheral nerves, motor neurons, and CNS, may be an important determinant of fatigue severity in this model. In human DM1, CUG^{exp} RNA sequesters MBNL proteins to form nuclear foci in neurons of the CNS and spinal cord,[55,56] suggesting that MBNL loss of function in these tissues also may contribute to fatigue in patients.

In the HSA^{LR} model, several lines of evidence suggest that progressive fatigue and its response to exercise training are independent of CUG^{exp} transcript abundance or alternative splicing patterns: (1) fatigue in saline-treated old HSA^{LR} mice was greater than in untreated young HSA^{LR} mice, yet CUG^{exp} RNA levels were significantly higher and alternative splicing mis-regulated to a significantly greater degree in young than in old mice, (2) fatigue persisted in mice treated with ASOs alone but was eliminated in mice that received combined exercise and ASO therapy, despite similar correction of the skeletal muscle molecular and histopathologic phenotype in each group, and (3) the molecular phenotype in muscle of the ASO-alone treatment group improved to a significantly greater degree than in the group that received exercise training alone, yet fatigue was worse in the ASO-alone treatment group. Unexpectedly, chronic exercise in HSA^{LR} mice reduces transcription of the *ACTA1*-CUG^{exp} transgene by a small degree, explaining the molecular observations in the exercise-alone group. By contrast, the quantity of *Dmpk*, the transcripts that carry the CUG^{exp} in DM1 patients, is unchanged in response to exercise, suggesting that translation of a potential benefit of exercise alone in DM1 patients would be independent of its effect on *DMPK*-CUG^{exp} RNA abundance.

Non-invasive monitoring of muscle size and composition is a potentially valuable tool for monitoring disease status, progression, and eventually response to therapy in future clinical trials.[57] EIM measurements are highly dependent on the electrode size, spacing between electrodes, size of muscle, and thickness of skin and subcutaneous fat.[58] In this study, we found that the EIM parameter transverse f_c was an accurate predictor of muscle fiber size in the HSA^{LR} and *Mbnl1*$^{-/-}$ models of DM1 and in WT controls, with f_c values inversely related to fiber size. We suspect that the bimodal and extended distribution of f_c for old WT mice is an artifact caused by thicker skin and/or the larger and more variable collection of adipose tissue located between the skin and proximal gastrocnemius muscle in some old mice as compared to young mice. Our results support the

exploration of EIM as a non-invasive means to monitor disease status and burden in muscle tissue of DM1 patients.

The *ACTA1*-CTG repeat length of approximately 185 that we observed by Southern blotting is considerably shorter than the 250 that was reported at the time the model was originally published[29] and suggests the possibility of germline contraction. In human DM1 muscle tissue, the allele length of the *DMPK*-CTG repeat can reach up to 3,000 to 5,000 by somatic expansion and is associated with fatigue, muscle wasting, and histopathologic fiber atrophy.[39] The stability of the CTG repeat that we observed in aged HSA^{LR} muscle tissue suggests that fatigue can progress independently of somatic expansion and may explain, in part, the absence of muscle wasting in this model.

MATERIALS AND METHODS

Experimental mice

The Massachusetts General Hospital Institutional Animal Care and Use Committee (IACUC) approved all mouse studies. The HSA^{LR} transgenic and *Mbnl1*$^{-/-}$ mouse models of DM1, both on the FVB/n background, were described previously.[29,30] Friend Virus B NIH Jackson (FVB/n) WT mice served as controls. Mice ranged from 2 to 18 months of age and were chosen randomly by genotype and stratified for sex to allow an approximately equal number of females and males.

Exercise-activity monitoring assay

We tested all mice at night or synchronized to a reverse light cycle (shifted 12-h:12-h dark:light) room so that at the time of testing, the mice were behaviorally most active. Mice were acclimatized to the test room, on the same light:dark cycle, for at least 1 h. We exercised mice using an adjustable variable speed belt treadmill (Exer 3/6; Columbus Instruments), up a 15-degree grade with acclimatization at 3 m/min for 2 min, acceleration from 3 m/min to 15 m/min (18-month-old mice) or 20 m/min (young and 15-month-old mice) over 1 min, followed by 15 m/min (old mice) or 20 m/min (young mice) for 10 min and a 1-min cool-down period of the speed gradually slowing to a stop, for a total regimen of 14 min (modified from a prior study[59]). We monitored mouse activity using individual chambers in sets of two, for 30 × 1 min intervals pre- and/or immediately post-treadmill exercise in an isolated room at the same time every day and with the same handler (Opto-Varimex 4 activity meter; Columbus Instruments). This system uses a grid of invisible infrared light beams that traverse the animal chamber front to back and left to right to monitor the position and movement of the mouse within an x-y-z plane. The sensor pairs consist of an infrared emitter bar with eight infrared beams and a matching detector bar. Every interruption of one of the optimal beams is registered as a count. Vertical counts are the total counts from the z-axis sensors. Vertical breaks are single-rearing events with 1 s between each event. Ambulatory time is the quantification of the total time spent making large ambulatory movement (s). Rest time is defined as the combined period of time without detection of movement (s). Stereotypic time is the quantification of total time spent on small rapid movements such as scratching, grooming, or other stereotypic non-ambulatory movements (s). The total distance traveled during the interval is recorded in centimeters (cm). For each parameter, we used average values of three separate trials on three separate days for comparison between groups.

Moderate-intensity exercise training regimen

We exercised mice using an adjustable variable-speed belt treadmill (Exer 3/6; Columbus Instruments) on a flat surface (no incline) at 3 m/min for 2 min, acceleration from 3 m/min to 11.5 m/min over 1 min, followed by 11.5 m/min for 30 min and a 1-min cool-down period of the speed gradually slowing to a stop, for a total regimen of 34 min, 6 days per week for 15 weeks.[38] We prepared the mice for the full regimen by using a 15-day acclimatization period at a speed of 11.5 m/min for 3 min and increased the duration by 2 min per day until reaching the target duration of 30 min per session at full speed. HSA^{LR} mice started the acclimatization procedure at age ~14–14.5 months and completed the training regimen at age ~17.5–18 months.

EIM

We measured electrical impedance at 41 frequencies from 1 kHz to 10 MHz in gastrocnemius muscles of mice in the prone position under general anesthesia (inhaled isoflurane) using the EIM1103 System (Myolex, San Francisco, CA, USA).[35] The examiner (J.L.) was blinded to treatment. After removal of hair (Nair), electrode gel and a tetrapolar electrode array were placed on the outside of the skin in an orientation to enable application of electric current parallel to the long axis of the muscle and longitudinal with respect to muscle-fiber orientation. This four-electrode method applies current between two outer electrodes and uses two inner electrodes to measure the impedance to current flow as the overall resistance (R) encountered in combination with the reactance (X), which is a capacitive property of temporary storage and release of electric current by muscle-fiber membranes. Values for both resistance and reactance are measured in ohms. The time shift of electric current that occurs as a result of its passage through muscle tissue is known as the phase angle (θ) and is calculated using the formula $\theta = \text{arctangent}(R/X)$.[34] Following data capture in the longitudinal orientation, the electrode array was rotated 90° so that the electrodes were oriented to enable application of current perpendicular to the long axis of the muscle and transverse with respect to muscle-fiber orientation for capture of a transverse dataset, then rotated an additional 90° (180° from dataset #1) for capture of a second longitudinal dataset, and finally rotated an additional 90° (270° from dataset #1) for capture of a second transverse dataset. We sampled the left and right gastrocnemius muscles with electrodes placed twice in the longitudinal direction and twice in the transverse direction and averaged the longitudinal and transverse sets for each muscle. To establish a relationship between the impedance data and muscle fiber morphometry and physiology, we used an empiric model proposed by Cole[60] that summarizes the frequency dependence of the measured resistance and reactance values into four parameters that relate to the electrical and geometrical properties of muscle fibers.[58,61] One of these parameters, the

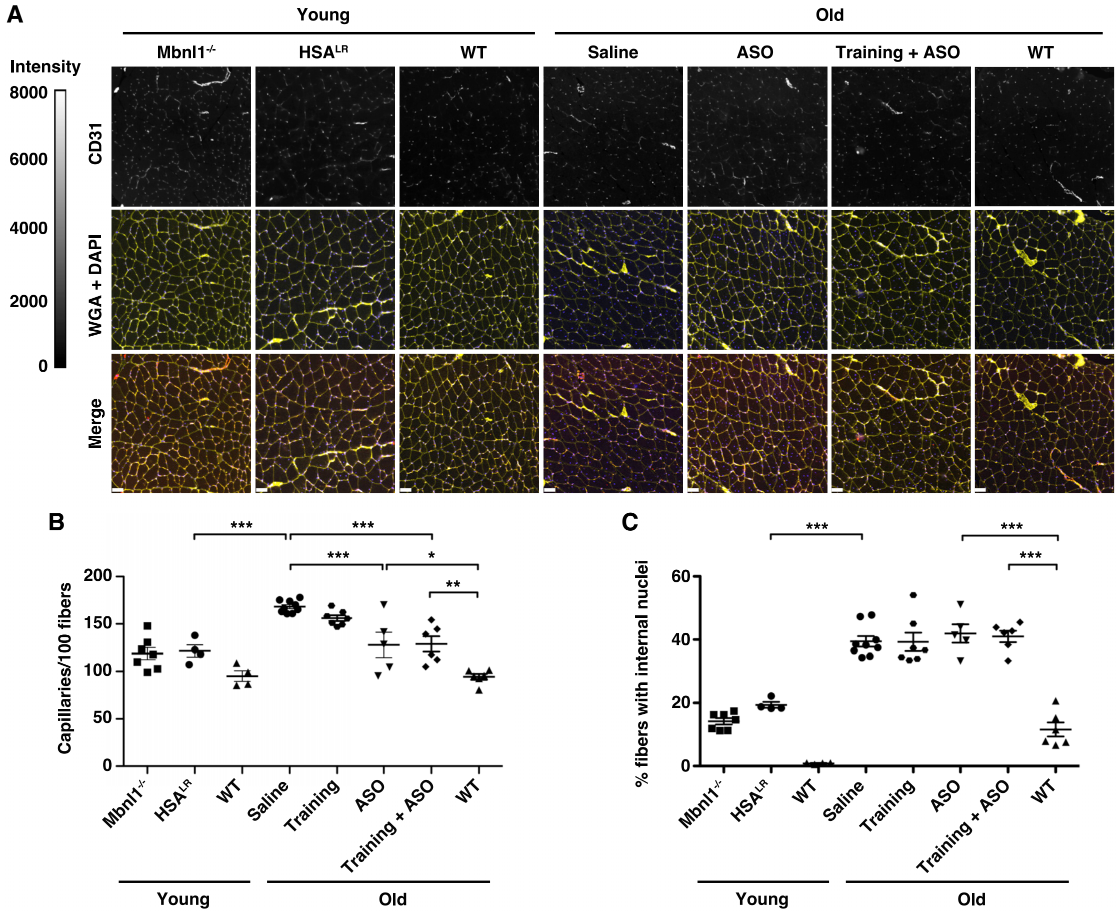

Figure 4. Age-related progression of myopathy and response to ASO treatment in DM1 mice with muscle fatigue

We immunolabeled capillaries with anti-CD31 antibody, outlined muscle fibers with Alexa 647-wheat germ agglutinin (WGA), and highlighted nuclei with DAPI. (A) Representative images of CD31, WGA+DAPI, and CD31+WGA+DAPI (merge) of gastrocnemius muscles from each group are shown. Fluorescence intensity range is 0–8,000 grayscale units. Bars, 50 μm. (B) Quantification of capillary density displayed as the number of capillaries per 100 muscle fibers. ***p < 0.001; **p < 0.01; *p < 0.05; one-way ANOVA/Tukey. (C) Quantification of the percentage of gastrocnemius muscle fibers in each group that contain at least one internal nucleus. ***p < 0.001; one-way ANOVA/Tukey. Error bars indicate mean ± SEM.

f_c, is the frequency of maximum reactance and is inversely related to muscle fiber size.

ASO treatment

We used ASO 445236, a 20-mer RNase H-active gapmer that targets *ACTA1* transcripts 3′ of the expanded CUG repeat region.[8] The central gap segment consists of ten 2′-deoxyribonucleotides (underlined in the sequence below) that are flanked on the 5′ and 3′ wings by five 2′-*O*-methoxyethyl-modified nucleotides. The internucleotide linkages are phosphorothioate, and all cytosine residues are 5′ methylcytosines. We treated mice with a dose of 25 mg/kg by subcutaneous injection twice weekly for 4 weeks (eight doses) and again once every 4 weeks (two additional doses for a total of ten doses). The ASO was a gift from C. Frank Bennett (Ionis Pharmaceuticals, Carlsbad, CA, USA). Treatment assignments of saline, ASO, or training regimen combined with ASO were randomized.

The gapmer ASO #445236 sequence is as follows: 5′-CCATT TTCTTCCACAGGGCT-3′ (published previously[8]).

Muscle fiber histology, immunolabeling, and morphometry

We stained 8-μm frozen sections with hematoxylin Gill #2 (Sigma product number GHS216) for 2 min, rinsed in running water until clear, 1× Scott's water (Sigma product number S5134) 1 min, and eosin Y (Sigma product number HT-110116) 2 min, rinsed in distilled water until the eosin stopped streaking, 50% ethanol for 1 min, 70% ethanol for 1 min, 95% ethanol for 1 min twice, 100% ethanol for 1 min twice, and xylenes (Fisher product number HC700) 1 min. Slides were let to dry and then mounted with Permount (Fisher product number SP15). To identify capillaries, we fixed 8-μm muscle-tissue sections with 3% paraformaldehyde (pH 7.3) for 10 min at room temperature, washed with PBS, incubated in anti-CD31 rabbit monoclonal primary antibody (5.5 μg/mL; Abcam product number EPR17260-263) overnight at 4°C,

followed by a goat anti-rabbit Alexa 546 secondary antibody (1 μg/mL; Invitrogen product number A11018) for 1 h at room temperature.[11] To highlight the muscle fibers for the measurement of minimum Feret's diameter, defined as the minimum distance between parallel tangents,[33] and counterstain nuclei, we added Alexa 647-wheat germ agglutinin (20 μg/mL; Invitrogen product number W32466) and DAPI (33 ng/mL) together with the secondary antibody. We mounted slides using anti-fade medium (Prolong Gold; Invitrogen product number P36930) and No. 1 1/2 cover glasses (Zeiss product number 474030-9000-000), as previously described.[11] In the gastrocnemius muscles, quantification of capillary density, internal nuclei, and fiber diameter was blinded to treatment assignment. In the paraspinal muscles, which were examined after the gastrocnemius, these measurements were unblinded.

To localize MBNL1 protein, we incubated muscle sections in anti-MBNL1 rabbit polyclonal antibody (2 μg/mL in PBS; Abcam product number ab45899) overnight at 4°C followed by goat anti-rabbit Alexa 546 secondary antibody (1 μg/mL in PBS) for 1 h at room temperature. To highlight muscle fibers and nuclei, we added fluorescein isothiocyanate (FITC)-wheat germ agglutinin (10 μg/mL; Sigma product number L4895) and DAPI (33 ng/mL) together with the secondary antibody.

Fluorescence *in situ* hybridization (FISH)

To identify CUG^{exp} RNA foci, we modified a previously published protocol.[62] We fixed 8-μm muscle cryosections with 3% paraformaldehyde (pH 7.3) in 1× PBS, washed in 1× PBS, permeabilized nuclei using 0.5% Triton X-100 in 1× PBS for 5 min or 0.2% Triton X-100 in 1× PBS for 10 min at room temperature, and incubated in pre-hybridization solution (30% formamide/2× saline sodium citrate [SSC]) for 10 min at room temperature. Hybridization was for 3 h at 37°C in 33% formamide, 2× SSC, 0.2 mg/mL bovine serum albumin (NEB product #B9001S), 70 μg/mL yeast tRNA (Invitrogen product #15401-011), 2 mM ribonucleoside vanadyl complex (NEB product #S1402S), and 1 μg/mL 2′-O-methyl-modified RNA CAG repeat probe 5′ labeled with Alexa 647. Post-hybridization, we incubated sections in 30% formamide/2× SSC for 30 min at 42°C followed by 1× SSC for 30 min at room temperature and three washes in 1× PBS at room temperature before proceeding to labeling with primary anti-MBNL1 antibody, as described above.

Hybridization probe

The hybridization probe is as follows: 5′-Alexa 647-mCmAmGmC mAmGmCmAmGmCmAmGmCmAmGmCmAmGmCmA-3′ (high-performance liquid chromatography [HPLC] purified; "m" designates that RNA bases have 2′-O-methyl modifications; IDT).

Light and quantitative fluorescence microscopy

To capture single images or z series stacks, we used an AxioImager microscope (Zeiss), filters for DAPI (excitation/emission 365/445; Zeiss filter set 49), GFP (excitation/emission 470/525; Zeiss filter set 38), Cy3 (excitation/emission 550/605; Zeiss filter set 43 HE), and Cy5 (excitation/emission 640/690; Zeiss filter set 50), a Flash 4.0 LT sCMOS camera (Hamamatsu), a MicroPublisher 3.3 RTV color CCD camera (Q-Imaging), and Volocity image acquisition software (PerkinElmer). Objectives were 5× EC Plan-NEOFLUAR NA 0.16, 10× EC Plan-NEOFLUAR NA 0.3, 20× Plan-APOCHROMAT NA 0.8, 40× Plan-APOCHROMAT NA 1.4, and 63× Plan-APOCHROMAT NA 1.4. To quantitate fluorescence, we used Volocity quantitation and restoration software modules (PerkinElmer), as described previously.[11]

RNA isolation

We homogenized muscle tissues in Trizol (Life Technologies), removed DNA and protein using bromochloropropane, precipitated RNA with isopropanol, washed pellets in 75% ethanol, and dissolved pellets in molecular grade water according to manufacturer recommendations. To determine RNA concentration and quality, we measured A260 and A280 values (Nanodrop) and examined 18S and 28S ribosomal RNA bands by agarose gel electrophoresis, as described previously.[11]

Quantification of gene expression by ddPCR

We used ddPCR supermix for probes (Bio-Rad product number 186-3010), an automated droplet generator (Bio-Rad QX200), automated droplet reader (Bio-Rad QX200), and PCR cycling conditions according to manufacturer instructions, as previously published.[40] To measure gene-expression level, we used a previously published primer pair and Fam-labeled probe for human *ACTA1* mRNA[6] and used Primer3 software[51,52] to design a primer pair and Fam-labeled probe targeting *ACTA1* pre-mRNA at the intron 1-exon 2 splice site. The primer pair and Fam-labeled probe that we used to measure mouse *Dmpk* was published previously.[8] The sequences are shown in Table S1. For normalization controls, we used commercially available standard assays for mouse *Acta1* (Hex-labeled probe; Bio-Rad unique assay ID dMmuCPE5088325) and mouse general transcription factor 2b (*Gtf2b*; FAM-MGB; Applied Biosciences assay ID Mm00663250_m1).[40] After PCR was complete, the plate was loaded into the droplet reader, processed, and analyzed using QuantaSoft software, and total events were quantitated using the mean copy number per μL of duplicate 20-μL assays from individual samples.[40]

RT-PCR analysis of alternative splicing

We made cDNA using Superscript III reverse transcriptase (Life Technologies) and random primers and performed PCR using Amplitaq Gold (Life Technologies) and gene-specific primers. We separated PCR products using agarose gels, labeled DNA with 1× SYBR I green nucleic acid gel stain (Life Technologies product number S7567)/1× Tris-borate-EDTA (TBE) for 1 h, and quantitated band intensities using a transilluminator, CCD camera, XcitaBlue conversion screen, and Image Lab image acquisition and analysis software (Bio-Rad), as previously described.[40] Sequences for primers targeting alternative splicing of *Atp2a1*, *Clcn1*, *Clasp1*, and *Titin* are shown in Table S2 and have been published previously.[11,31,44]

Estimation of the *ACTA1* transgene CTG repeat length

We digested 100 ng genomic DNA isolated from tail-biopsy DNA at weaning (3 weeks of age) and from gastrocnemius or extensor digitorum brevis muscles collected at ~18 months of age with the restriction enzyme HindIII-HF (NEB product number 3104) and used 10 ng of digested DNA as template for PCR with primers spanning the CTG repeat insertion (Figure S5; Table S2). To estimate size, we separated

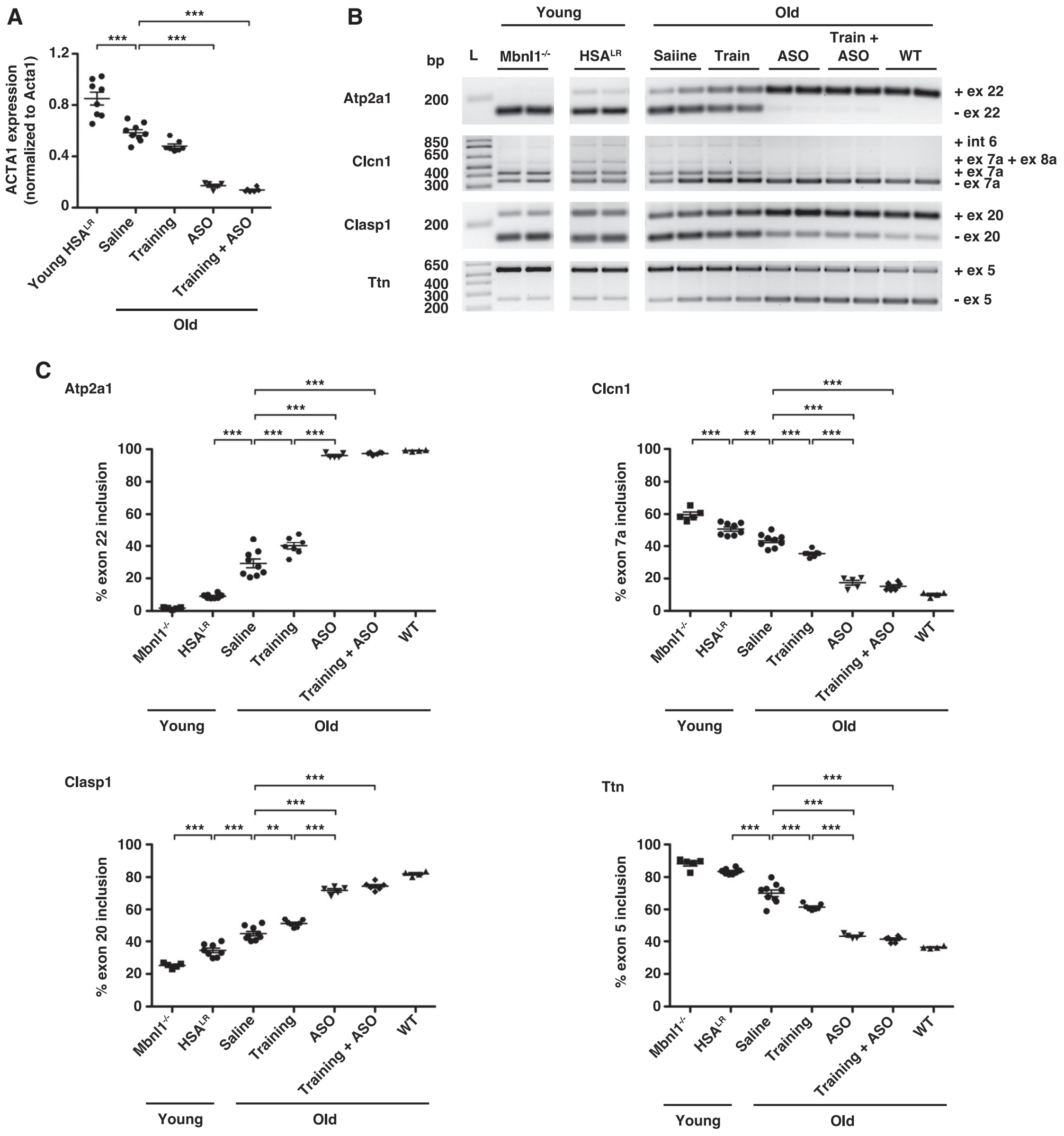

Figure 5. Muscle fatigue is independent of molecular disease activity in old DM1 mice

We treated old HSA^{LR} mice (~14 months of age) with saline (N = 9), moderate-exercise training program (N = 7; training; see Materials and Methods), ASO 445236 (N = 5), or a combination of training and ASO 445236 (N = 6) for 3.5 months and analyzed at ~18 months of age. Young HSA^{LR} mice (N = 8) served as controls. (A) ddPCR quantification of *ACTA1*-CUG^{exp} transcripts in gastrocnemius muscle normalized to endogenous mouse *Acta1* transcripts. ***p < 0.001; one-way ANOVA/Tukey. (B) RT-PCR and agarose gel electrophoresis analysis of alternative splicing of transcripts *Atp2a1*, *Clasp1*, *Clcn1*, and *Ttn* in gastrocnemius muscles of young and old HSA^{LR} mice. Gastrocnemius muscles of young *Mbnl1*$^{-/-}$ and old WT mice served as controls. (C) Quantification of alternative splicing in gastrocnemius muscles. ***p < 0.001; **p < 0.01; one-way ANOVA/Tukey. Error bars indicate mean ± SEM.

PCR products by 1% agarose gel electrophoresis, stained gels using 1× SYBR I green, and imaged PCR products as described above.

Small-pool PCR and Southern blotting

We also examined CTG repeat length by small-pool PCR and Southern blotting,[50,63] with modifications as follows. We used 2 ng of HindIII-HF-digested genomic DNA as template for PCR, separated products using 1% agarose gels, denatured using 0.5 M NaOH/1.5 M NaCl for 60 min with gentle shaking at room temperature, neutralized with 10× SSC for 30 min with gentle shaking at room temperature, and transferred DNA to nylon+ membranes overnight by capillary action using 10× SSC buffer. The next day, we cross-linked the DNA to the

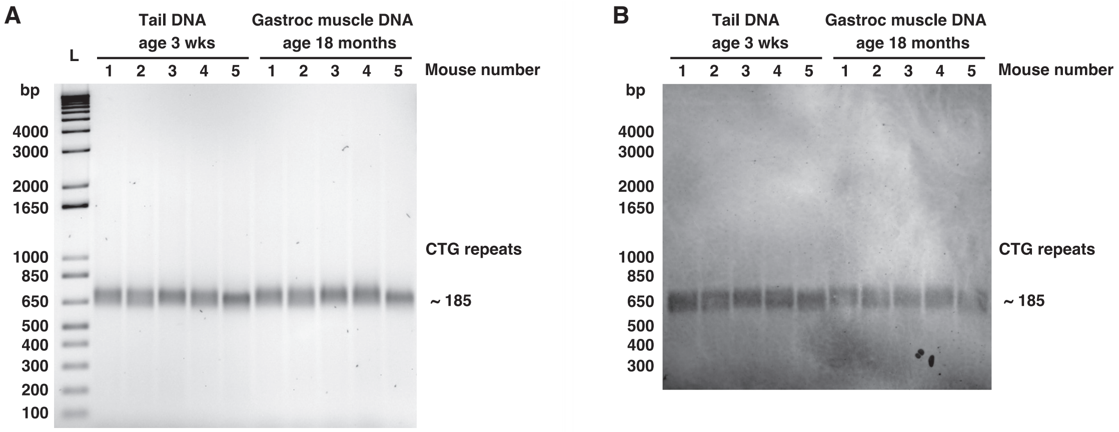

Figure 6. Progressive fatigue occurs without somatic expansion of the CTG repeat length in muscle tissue

We examined CTG repeat length in tail biopsy DNA obtained from HSA^{LR} mice at 3 weeks of age (N = 5; left half of gel) and in gastrocnemius (gastroc) muscle DNA obtained from the same mice at age 18 months using PCR and primers targeting the *ACTA1* sequence spanning the CTG insertion site. (A) PCR using 10-ng template DNA, gel electrophoresis, and staining with 1× SYBR green. bp, base pairs; L = 1 Kb Plus DNA ladder. CTG repeat length is estimated by subtracting the number of nucleotides amplified by the primers upstream and downstream of the CTG repeat from the total amplicon size of ~650 bp and dividing by 3 (see Figure S6). (B) Southern blot using 2 ng template DNA for PCR of the same samples in (A).

membrane using ultraviolet light (Stratalinker 1800; Stratagene), prehybridized membranes using ExpressHyb hybridization solution (Clontech product number 8015-3) for 30 min at 42°C, hybridized using a 5′-digoxigenin-labeled CAG repeat probe (50 ng/mL) in ExpressHyb for 1 h at 42°C, and washed in 2× SSC/0.1% sodium dodecyl sulfate (SDS) for 30 min at room temperature followed by 0.1× SSC/0.1% SDS for 30 min at 42°C. A rotating hybridization oven was used for the pre-hybridization, hybridization, and second post-hybridization wash steps; an orbital shaker was used for the first wash step. After the hybridization step, we washed membranes in 2× SSC/0.1% SDS for 15 min × 2 followed by 0.1× SSC/0.1% SDS for 15 min × 2, blocked with 0.6% gelatin from cold water fish skin[64] (Sigma product number G7041) in 1× PBS for 1 h at room temperature, and placed in mouse monoclonal anti-digoxigenin primary antibody immunoglobulin G (IgG)1kappa (clone 1.71.256; Sigma product number 11333062910) stock concentration 100 μg/mL (use concentration of 1:5,000 = 20 ng/mL PBS/0.05% Tween 20 for 1 h at room temperature in a rotating oven) and then overnight at 4°C. We used secondary antibody goat anti-mouse IRDye 680D (75 ng/mL; LI-COR product number 926-68070) in PBS/0.05% Tween 20 for 1 h at room temperature and imaged blots using a laser scanner (LI-COR Odyssey Cxl) and ImageStudio software (LI-COR).

Hybridization probe

The hybridization probe is as follows: 5′-DigN-mCmAmGmC-mAmGmCmAmGmCmAmGmCmAmGmCmAmGmCmA-3′ (HPLC purified; "m" designates that RNA bases have 2′-O-methyl modifications; IDT).

Sample size

Whether HSA^{LR} or *Mbnl1*$^{-/-}$ mice had exaggerated fatigue or differential EIM parameters relative to WT was unknown. Therefore, we were unable to choose a sample size ahead of time to ensure adequate power to measure response to therapy. Instead, we chose sample sizes based on previously reported differences of the molecular phenotype and/or response to ASO therapy in muscle tissue of these models,[8,11] combined with prior EIM experience using other mouse models.[35,65]

Statistical analysis

For two-group and multi-group comparisons, we used unpaired two-tailed t test or analysis of variance (ANOVA), respectively (Prism software, GraphPad). Group data are presented as mean ± SEM. A p value <0.05 was considered significant.

Data availability

All relevant data are available from the authors.

SUPPLEMENTAL INFORMATION

Supplemental Information can be found online at https://doi.org/10.1016/j.omtn.2020.11.014.

ACKNOWLEDGMENTS

The authors thank C. Neville for access and use of a hybridization oven, V. Comaills for assistance with the Odyssey imager, J. Nagy for assistance with EIM data analysis, C.F. Bennett for providing the ASO used in this study, and J.A. Sizemore for producing the graphical abstract. The Elaine and Richard Slye Fund (T.M.W.), Muscular Dystrophy Association (award ID 234905; T.M.W.), and the National Institutes of Health (NS088202; T.M.W.) supported the work.

AUTHOR CONTRIBUTIONS

N.H., E.K., L.A., J.L., P.G.-P., and T.M.W. performed experiments and analyzed data. S.B.R. analyzed data. T.M.W. designed the study and

wrote the paper. N.H., E.K., L.A., J.L., P.G.-P., S.B.R., and T.M.W. discussed the results and commented on the manuscript.

DECLARATION OF INTERESTS

S.B.R. serves as a consultant, scientific advisor, and board member and has equity in Myolex, a company that designs impedance devices for clinical and research use. S.B.R. is named an inventor of patented impedance technology that Myolex has the option to license.

REFERENCES

1. Harper, P.S. (1989). Myotonic Dystrophy, Second Edition (W.B. Saunders).
2. Ashizawa, T., Gagnon, C., Groh, W.J., Gutmann, L., Johnson, N.E., Meola, G., Moxley, R., 3rd, Pandya, S., Rogers, M.T., Simpson, E., et al. (2018). Consensus-based care recommendations for adults with myotonic dystrophy type 1. Neurol. Clin. Pract. *8*, 507–520.
3. Brook, J.D., McCurrach, M.E., Harley, H.G., Buckler, A.J., Church, D., Aburatani, H., Hunter, K., Stanton, V.P., Thirion, J.P., Hudson, T., et al. (1992). Molecular basis of myotonic dystrophy: expansion of a trinucleotide (CTG) repeat at the 3′ end of a transcript encoding a protein kinase family member. Cell *68*, 799–808.
4. Harley, H.G., Rundle, S.A., MacMillan, J.C., Myring, J., Brook, J.D., Crow, S., Reardon, W., Fenton, I., Shaw, D.J., and Harper, P.S. (1993). Size of the unstable CTG repeat sequence in relation to phenotype and parental transmission in myotonic dystrophy. Am. J. Hum. Genet. *52*, 1164–1174.
5. Overend, G., Légaré, C., Mathieu, J., Bouchard, L., Gagnon, C., and Monckton, D.G. (2019). Allele length of the DMPK CTG repeat is a predictor of progressive myotonic dystrophy type 1 phenotypes. Hum. Mol. Genet. *28*, 2245–2254.
6. Wheeler, T.M., Sobczak, K., Lueck, J.D., Osborne, R.J., Lin, X., Dirksen, R.T., and Thornton, C.A. (2009). Reversal of RNA dominance by displacement of protein sequestered on triplet repeat RNA. Science *325*, 336–339.
7. Mulders, S.A., van den Broek, W.J., Wheeler, T.M., Croes, H.J., van Kuik-Romeijn, P., de Kimpe, S.J., Furling, D., Platenburg, G.J., Gourdon, G., Thornton, C.A., et al. (2009). Triplet-repeat oligonucleotide-mediated reversal of RNA toxicity in myotonic dystrophy. Proc. Natl. Acad. Sci. USA *106*, 13915–13920.
8. Wheeler, T.M., Leger, A.J., Pandey, S.K., MacLeod, A.R., Nakamori, M., Cheng, S.H., Wentworth, B.M., Bennett, C.F., and Thornton, C.A. (2012). Targeting nuclear RNA for in vivo correction of myotonic dystrophy. Nature *488*, 111–115.
9. Bisset, D.R., Stepniak-Konieczna, E.A., Zavaljevski, M., Wei, J., Carter, G.T., Weiss, M.D., and Chamberlain, J.R. (2015). Therapeutic impact of systemic AAV-mediated RNA interference in a mouse model of myotonic dystrophy. Hum. Mol. Genet. *24*, 4971–4983.
10. Pinto, B.S., Saxena, T., Oliveira, R., Méndez-Gómez, H., Cleary, J.D., Denes, L.T., McConnell, O., Arboleda, J., Xia, G., Swanson, M.S., and Wang, E.T. (2017). Impeding Transcription of Expanded Microsatellite Repeats by Deactivated Cas9. Mol. Cell *68*, 479–490.e5.
11. Hu, N., Antoury, L., Baran, T.M., Mitra, S., Bennett, C.F., Rigo, F., Foster, T.H., and Wheeler, T.M. (2018). Non-invasive monitoring of alternative splicing outcomes to identify candidate therapies for myotonic dystrophy type 1. Nat. Commun. *9*, 5227.
12. Angelbello, A.J., Rzuczek, S.G., Mckee, K.K., Chen, J.L., Olafson, H., Cameron, M.D., Moss, W.N., Wang, E.T., and Disney, M.D. (2019). Precise small-molecule cleavage of an r(CUG) repeat expansion in a myotonic dystrophy mouse model. Proc. Natl. Acad. Sci. USA *116*, 7799–7804.
13. Heatwole, C., Bode, R., Johnson, N., Quinn, C., Martens, W., McDermott, M.P., Rothrock, N., Thornton, C., Vickrey, B., Victorson, D., and Moxley, R., 3rd (2012). Patient-reported impact of symptoms in myotonic dystrophy type 1 (PRISM-1). Neurology *79*, 348–357.
14. Kimura, J. (2013). Electrodiagnosis in Diseases of Nerve and Muscle, Fourth Edition (Oxford University Press).
15. Chaudhuri, A., and Behan, P.O. (2004). Fatigue in neurological disorders. Lancet *363*, 978–988.
16. Kalkman, J.S., Schillings, M.L., van der Werf, S.P., Padberg, G.W., Zwarts, M.J., van Engelen, B.G., and Bleijenberg, G. (2005). Experienced fatigue in facioscapulohumeral dystrophy, myotonic dystrophy, and HMSN-I. J. Neurol. Neurosurg. Psychiatry *76*, 1406–1409.
17. Moyer, M., Berger, D.S., Ladd, A.N., and Van Lunteren, E. (2011). Differential susceptibility of muscles to myotonia and force impairment in a mouse model of myotonic dystrophy. Muscle Nerve *43*, 818–827.
18. Esposito, F., Cè, E., Rampichini, S., Monti, E., Limonta, E., Fossati, B., and Meola, G. (2017). Electromechanical delays during a fatiguing exercise and recovery in patients with myotonic dystrophy type 1. Eur. J. Appl. Physiol. *117*, 551–566.
19. Angelini, C., and Tasca, E. (2012). Fatigue in muscular dystrophies. Neuromuscul. Disord. *22* (*Suppl 3*), S214–S220.
20. Zwarts, M.J., and van Weerden, T.W. (1989). Transient paresis in myotonic syndromes. A surface EMG study. Brain *112*, 665–680.
21. Torres, C., Moxley, R.T., and Griggs, R.C. (1983). Quantitative testing of handgrip strength, myotonia, and fatigue in myotonic dystrophy. J. Neurol. Sci. *60*, 157–168.
22. van der Werf, S., Kalkman, J., Bleijenberg, G., van Engelen, B., Schillings, M., and Zwarts, M.; Netherlands Fatigue Research Group (2003). The relation between daytime sleepiness, fatigue, and reduced motivation in patients with adult onset myotonic dystrophy. J. Neurol. Neurosurg. Psychiatry *74*, 138–139.
23. Hamel, J., Johnson, N., Tawil, R., Martens, W.B., Dilek, N., McDermott, M.P., and Heatwole, C. (2019). Patient-Reported Symptoms in Facioscapulohumeral Muscular Dystrophy (PRISM-FSHD). Neurology *93*, e1180–e1192.
24. Voet, N., Bleijenberg, G., Hendriks, J., de Groot, I., Padberg, G., van Engelen, B., and Geurts, A. (2014). Both aerobic exercise and cognitive-behavioral therapy reduce chronic fatigue in FSHD: an RCT. Neurology *83*, 1914–1922.
25. Larun, L., Brurberg, K.G., Odgaard-Jensen, J., and Price, J.R. (2019). Exercise therapy for chronic fatigue syndrome. Cochrane Database Syst. Rev. *10*, CD003200.
26. Grazioli, E., Tranchita, E., Borriello, G., Cerulli, C., Minganti, C., and Parisi, A. (2019). The Effects of Concurrent Resistance and Aerobic Exercise Training on Functional Status in Patients with Multiple Sclerosis. Curr. Sports Med. Rep. *18*, 452–457.
27. Sheehan, P., Denieffe, S., Murphy, N.M., and Harrison, M. (2020). Exercise is more effective than health education in reducing fatigue in fatigued cancer survivors. Support. Care Cancer *28*, 4953–4962.
28. Voet, N.B., van der Kooi, E.L., van Engelen, B.G., and Geurts, A.C. (2019). Strength training and aerobic exercise training for muscle disease. Cochrane Database Syst. Rev. *12*, CD003907.
29. Mankodi, A., Logigian, E., Callahan, L., McClain, C., White, R., Henderson, D., Krym, M., and Thornton, C.A. (2000). Myotonic dystrophy in transgenic mice expressing an expanded CUG repeat. Science *289*, 1769–1773.
30. Kanadia, R.N., Johnstone, K.A., Mankodi, A., Lungu, C., Thornton, C.A., Esson, D., Timmers, A.M., Hauswirth, W.W., and Swanson, M.S. (2003). A muscleblind knockout model for myotonic dystrophy. Science *302*, 1978–1980.
31. Lin, X., Miller, J.W., Mankodi, A., Kanadia, R.N., Yuan, Y., Moxley, R.T., Swanson, M.S., and Thornton, C.A. (2006). Failure of MBNL1-dependent post-natal splicing transitions in myotonic dystrophy. Hum. Mol. Genet. *15*, 2087–2097.
32. Du, H., Cline, M.S., Osborne, R.J., Tuttle, D.L., Clark, T.A., Donohue, J.P., Hall, M.P., Shiue, L., Swanson, M.S., Thornton, C.A., and Ares, M., Jr. (2010). Aberrant alternative splicing and extracellular matrix gene expression in mouse models of myotonic dystrophy. Nat. Struct. Mol. Biol. *17*, 187–193.
33. Briguet, A., Courdier-Fruh, I., Foster, M., Meier, T., and Magyar, J.P. (2004). Histological parameters for the quantitative assessment of muscular dystrophy in the mdx-mouse. Neuromuscul. Disord. *14*, 675–682.
34. Rutkove, S.B. (2009). Electrical impedance myography: Background, current state, and future directions. Muscle Nerve *40*, 936–946.
35. Li, J., Pacheck, A., Sanchez, B., and Rutkove, S.B. (2016). Single and modeled multifrequency electrical impedance myography parameters and their relationship to force production in the ALS SOD1G93A mouse. Amyotroph. Lateral Scler. Frontotemporal Degener. *17*, 397–403.
36. Nagy, J.A., Kapur, K., Taylor, R.S., Sanchez, B., and Rutkove, S.B. (2018). Electrical impedance myography as a biomarker of myostatin inhibition with ActRIIB-mFc: a study in wild-type mice. Future Sci. OA *4*, FSO308.

37. Pandey, S.K., Wheeler, T.M., Justice, S.L., Kim, A., Younis, H.S., Gattis, D., Jauvin, D., Puymirat, J., Swayze, E.E., Freier, S.M., et al. (2015). Identification and characterization of modified antisense oligonucleotides targeting DMPK in mice and nonhuman primates for the treatment of myotonic dystrophy type 1. J. Pharmacol. Exp. Ther. *355*, 329–340.

38. Shefer, G., Rauner, G., Stuelsatz, P., Benayahu, D., and Yablonka-Reuveni, Z. (2013). Moderate-intensity treadmill running promotes expansion of the satellite cell pool in young and old mice. FEBS J. *280*, 4063–4073.

39. Nakamori, M., Sobczak, K., Puwanant, A., Welle, S., Eichinger, K., Pandya, S., Dekdebrun, J., Heatwole, C.R., McDermott, M.P., Chen, T., et al. (2013). Splicing biomarkers of disease severity in myotonic dystrophy. Ann. Neurol. *74*, 862–872.

40. Antoury, L., Hu, N., Balaj, L., Das, S., Georghiou, S., Darras, B., Clark, T., Breakefield, X.O., and Wheeler, T.M. (2018). Analysis of extracellular mRNA in human urine reveals splice variant biomarkers of muscular dystrophies. Nat. Commun. *9*, 3906.

41. Kimura, T., Nakamori, M., Lueck, J.D., Pouliquin, P., Aoike, F., Fujimura, H., Dirksen, R.T., Takahashi, M.P., Dulhunty, A.F., and Sakoda, S. (2005). Altered mRNA splicing of the skeletal muscle ryanodine receptor and sarcoplasmic/endoplasmic reticulum Ca2+-ATPase in myotonic dystrophy type 1. Hum. Mol. Genet. *14*, 2189–2200.

42. Mankodi, A., Takahashi, M.P., Jiang, H., Beck, C.L., Bowers, W.J., Moxley, R.T., Cannon, S.C., and Thornton, C.A. (2002). Expanded CUG repeats trigger aberrant splicing of ClC-1 chloride channel pre-mRNA and hyperexcitability of skeletal muscle in myotonic dystrophy. Mol. Cell *10*, 35–44.

43. Charlet-B, N., Savkur, R.S., Singh, G., Philips, A.V., Grice, E.A., and Cooper, T.A. (2002). Loss of the muscle-specific chloride channel in type 1 myotonic dystrophy due to misregulated alternative splicing. Mol. Cell *10*, 45–53.

44. Wheeler, T.M., Lueck, J.D., Swanson, M.S., Dirksen, R.T., and Thornton, C.A. (2007). Correction of ClC-1 splicing eliminates chloride channelopathy and myotonia in mouse models of myotonic dystrophy. J. Clin. Invest. *117*, 3952–3957.

45. Wagner, S.D., Struck, A.J., Gupta, R., Farnsworth, D.R., Mahady, A.E., Eichinger, K., Thornton, C.A., Wang, E.T., and Berglund, J.A. (2016). Dose-Dependent Regulation of Alternative Splicing by MBNL Proteins Reveals Biomarkers for Myotonic Dystrophy. PLoS Genet. *12*, e1006316.

46. Anvret, M., Ahlberg, G., Grandell, U., Hedberg, B., Johnson, K., and Edström, L. (1993). Larger expansions of the CTG repeat in muscle compared to lymphocytes from patients with myotonic dystrophy. Hum. Mol. Genet. *2*, 1397–1400.

47. Thornton, C.A., Johnson, K., and Moxley, R.T., 3rd (1994). Myotonic dystrophy patients have larger CTG expansions in skeletal muscle than in leukocytes. Ann. Neurol. *35*, 104–107.

48. Wong, L.J., Ashizawa, T., Monckton, D.G., Caskey, C.T., and Richards, C.S. (1995). Somatic heterogeneity of the CTG repeat in myotonic dystrophy is age and size dependent. Am. J. Hum. Genet. *56*, 114–122.

49. Zatz, M., Passos-Bueno, M.R., Cerqueira, A., Marie, S.K., Vainzof, M., and Pavanello, R.C. (1995). Analysis of the CTG repeat in skeletal muscle of young and adult myotonic dystrophy patients: when does the expansion occur? Hum. Mol. Genet. *4*, 401–406.

50. Monckton, D.G., Wong, L.J., Ashizawa, T., and Caskey, C.T. (1995). Somatic mosaicism, germline expansions, germline reversions and intergenerational reductions in myotonic dystrophy males: small pool PCR analyses. Hum. Mol. Genet. *4*, 1–8.

51. U.S. Department of Health and Human Services (2018). Physical Activity Guidelines for Americans, Second Edition (U.S. Department of Health and Human Services), https://health.gov/sites/default/files/2019-09/Physical_Activity_Guidelines_2nd_edition.pdf.

52. Canadian Society for Exercise Physiology (2020). Canadian Physical Activity Guidelines (Canadian Society for Exercise Physiology), https://csep.ca/CMFiles/Guidelines/CSEP_PAGuidelines_0-65plus_en.pdf.

53. Department of Health and Social Care (2019). UK Chief Medical Officers' Physical Activity Guidelines (Department of Health and Social Care), https://assets.publishing.service.gov.uk/government/uploads/system/uploads/attachment_data/file/832868/uk-chief-medical-officers-physical-activity-guidelines.pdf.

54. European Union Member State Sport Ministers (2008). European Union Physical Activity Guidelines (European Union Member State Sport Ministers), https://eacea.ec.europa.eu/sites/eacea-site/files/eu-physical-activity-guidelines-2008.pdf.

55. Jiang, H., Mankodi, A., Swanson, M.S., Moxley, R.T., and Thornton, C.A. (2004). Myotonic dystrophy type 1 is associated with nuclear foci of mutant RNA, sequestration of muscleblind proteins and deregulated alternative splicing in neurons. Hum. Mol. Genet. *13*, 3079–3088.

56. Wheeler, T.M., Krym, M.C., and Thornton, C.A. (2007). Ribonuclear foci at the neuromuscular junction in myotonic dystrophy type 1. Neuromuscul. Disord. *17*, 242–247.

57. Rutkove, S.B., Kapur, K., Zaidman, C.M., Wu, J.S., Pasternak, A., Madabusi, L., Yim, S., Pacheck, A., Szelag, H., Harrington, T., and Darras, B.T. (2017). Electrical impedance myography for assessment of Duchenne muscular dystrophy. Ann. Neurol. *81*, 622–632.

58. Sanchez, B., and Rutkove, S.B. (2017). Electrical Impedance Myography and Its Applications in Neuromuscular Disorders. Neurotherapeutics *14*, 107–118.

59. Kobayashi, Y.M., Rader, E.P., Crawford, R.W., Iyengar, N.K., Thedens, D.R., Faulkner, J.A., Parikh, S.V., Weiss, R.M., Chamberlain, J.S., Moore, S.A., and Campbell, K.P. (2008). Sarcolemma-localized nNOS is required to maintain activity after mild exercise. Nature *456*, 511–515.

60. Cole, K.S. (1940). Permeability and impermeability of cell membranes for ions. Cold Spring Harb. Symp. Quant. Biol. *8*, 110–122.

61. Arnold, W.D., Taylor, R.S., Li, J., Nagy, J.A., Sanchez, B., and Rutkove, S.B. (2017). Electrical impedance myography detects age-related muscle change in mice. PLoS ONE *12*, e0185614.

62. Mankodi, A., Urbinati, C.R., Yuan, Q.P., Moxley, R.T., Sansone, V., Krym, M., Henderson, D., Schalling, M., Swanson, M.S., and Thornton, C.A. (2001). Muscleblind localizes to nuclear foci of aberrant RNA in myotonic dystrophy types 1 and 2. Hum. Mol. Genet. *10*, 2165–2170.

63. Southern, E. (2006). Southern blotting. Nat. Protoc. *1*, 518–525.

64. Zavala, A.G., Kulkarni, A.S., and Fortunato, E.A. (2014). A dual color Southern blot to visualize two genomes or genic regions simultaneously. J. Virol. Methods *198*, 64–68.

65. Sanchez, B., Li, J., Yim, S., Pacheck, A., Widrick, J.J., and Rutkove, S.B. (2015). Evaluation of Electrical Impedance as a Biomarker of Myostatin Inhibition in Wild Type and Muscular Dystrophy Mice. PLoS ONE *10*, e0140521.

OMTN, Volume *23*

Supplemental Information

Antisense oligonucleotide and adjuvant exercise therapy reverse fatigue in old mice with myotonic dystrophy

Ningyan Hu, Eunjoo Kim, Layal Antoury, Jia Li, Paloma González-Pérez, Seward B. Rutkove, and Thurman M. Wheeler

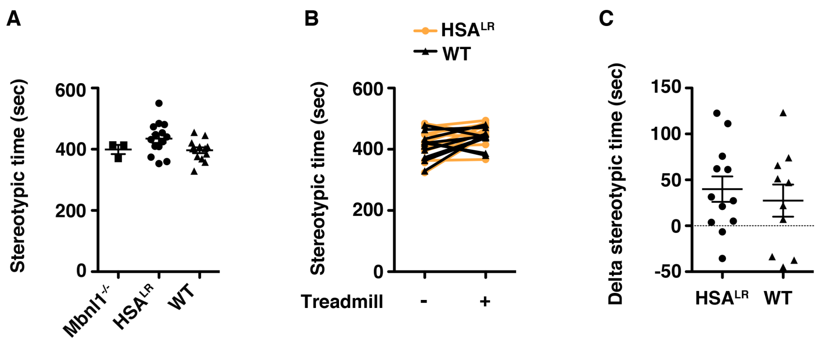

Figure S1. Spontaneous activity in two mouse models of DM1. We examined spontaneous x-, y-, and z-plane activity of untreated mice for 30 minutes in the dark either at night or in a reverse light cycle room, when mice are more active. (A) Quantitative activity measurements of the total time spent on small rapid movements such as scratching, grooming, or other stereotypic non-ambulatory movements (stereotypic time; seconds) during the 30 minute (1800-second) monitoring period in 2 - 3 month-old Mbnl1 knockout (-/-; N = 3), HSA^{LR} (N = 14), and wild-type (N = 13) immediately following treadmill exercise running for 10 minutes uphill at a 15 degree incline (see Materials and Methods). (B) Stereotypic time in 15 month-old HSA^{LR} (N = 12; orange circles) and WT (N = 10; black triangles) mice pre (-) and post (+) treadmill exercise running for 10 minutes uphill at a 15 degree incline. (C) The difference (delta) between the pre- and post-exercise measurements of each individual mouse shown in (B). Error bars indicate mean ± SEM. See also Figures 1 and 2.

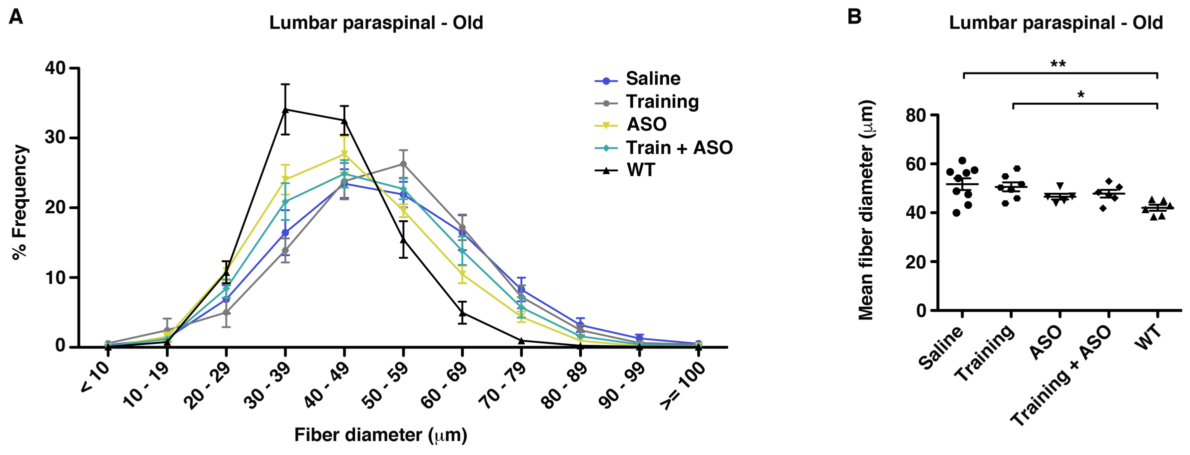

Figure S2. Relationship of fatigue in DM1 mice to muscle fiber diameter. We treated old HSA^{LR} mice (~ 14 months of age) with saline (N = 9), moderate intensity exercise training program (N = 7; Training; see Materials and Methods), ASO 445236 (N = 5), or a combination of training and ASO 445236 (N = 6; Train + ASO) for 3 ½ months and analyzed at ~ 18 months of age. Old wild type (WT; ~ 18 months of age) (N = 6) served as controls. (A) Minimum Feret's muscle fiber diameter, defined as the minimum distance between parallel tangents, in 8 μm cryosections of lumbar paraspinal muscles in each group. (B) Mean fiber diameter in each group using the data in a). ** $P < 0.01$; * $P = 0.012$; one-way ANOVA/Tukey. Error bars indicate mean ± SEM. See also Figure 3.

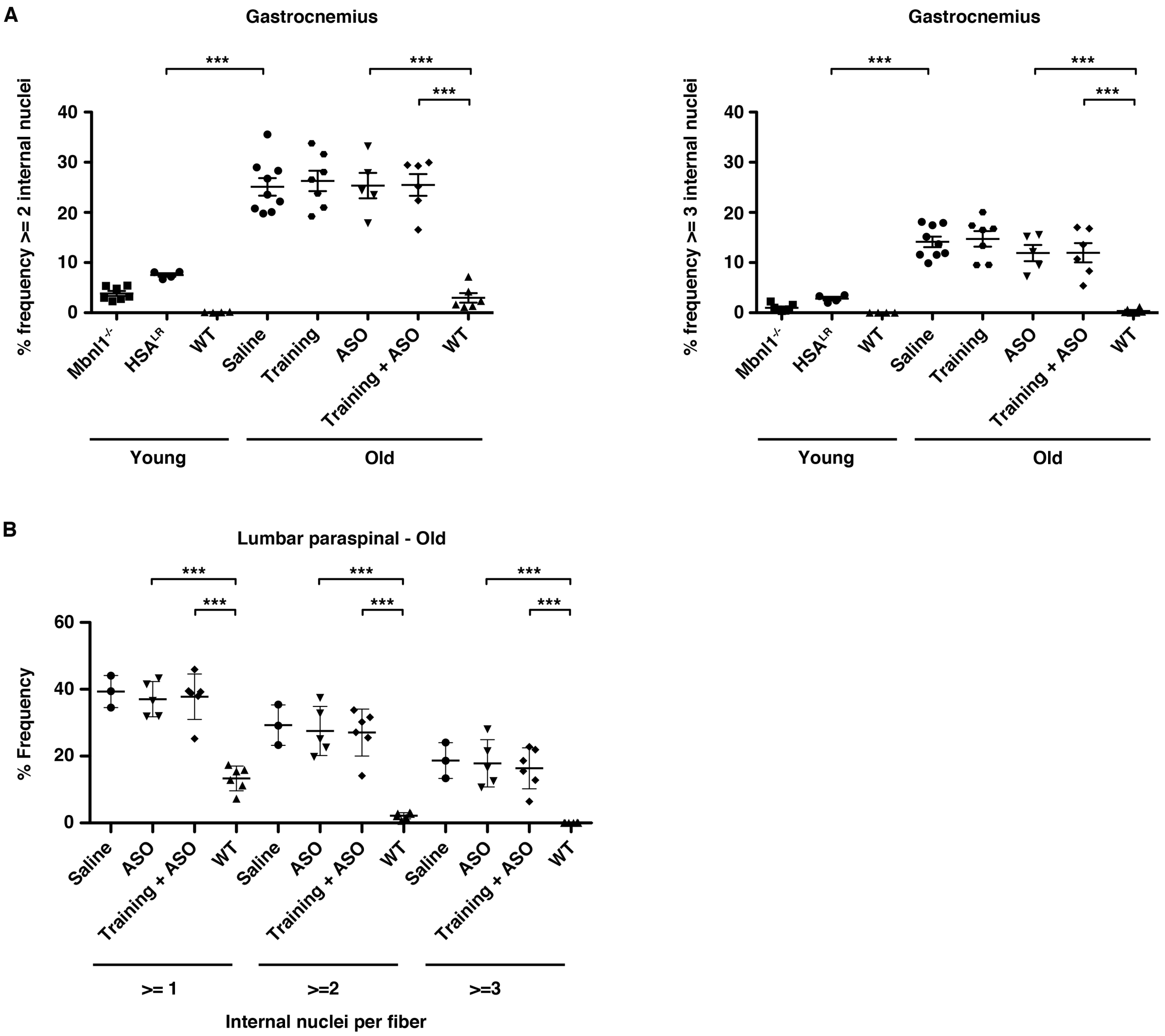

Figure S3. Relationship of fatigue to muscle histopathology. We treated old HSA^{LR} mice (~ 14 months of age) with saline (N = 9), a moderate intensity exercise training program (N = 7; Training; see Materials and Methods), ASO 445236 (N = 5), or a combination of training and ASO 445236 (N = 6) for 3 ½ months and analyzed at ~ 18 months of age. Old wild type (WT; ~ 18 months of age) (N = 6) served as controls. Muscle fibers were outlined with Alexa 647-wheat germ agglutinin (WGA) and nuclei highlighted with DAPI (see Fig 4). (A) Quantification of the percentage of gastrocnemius muscle fibers in each group that contain at least two internal nuclei (left) and at least three internal nuclei (right). Young (2 - 4 months of age) Mbnl1$^{-/-}$ (N = 6) and HSA^{LR} mice (N = 8) served as controls. *** $P < 0.001$; one-way ANOVA/Tukey. (B) Quantification of the percentage of lumbar paraspinal muscle fibers in old HSA^{LR} mice that contain at least one internal nucleus, at least two, or at least three internal nuclei. *** $P < 0.001$; one-way ANOVA/Tukey. Error bars indicate mean ± SEM. See also Figure 4.

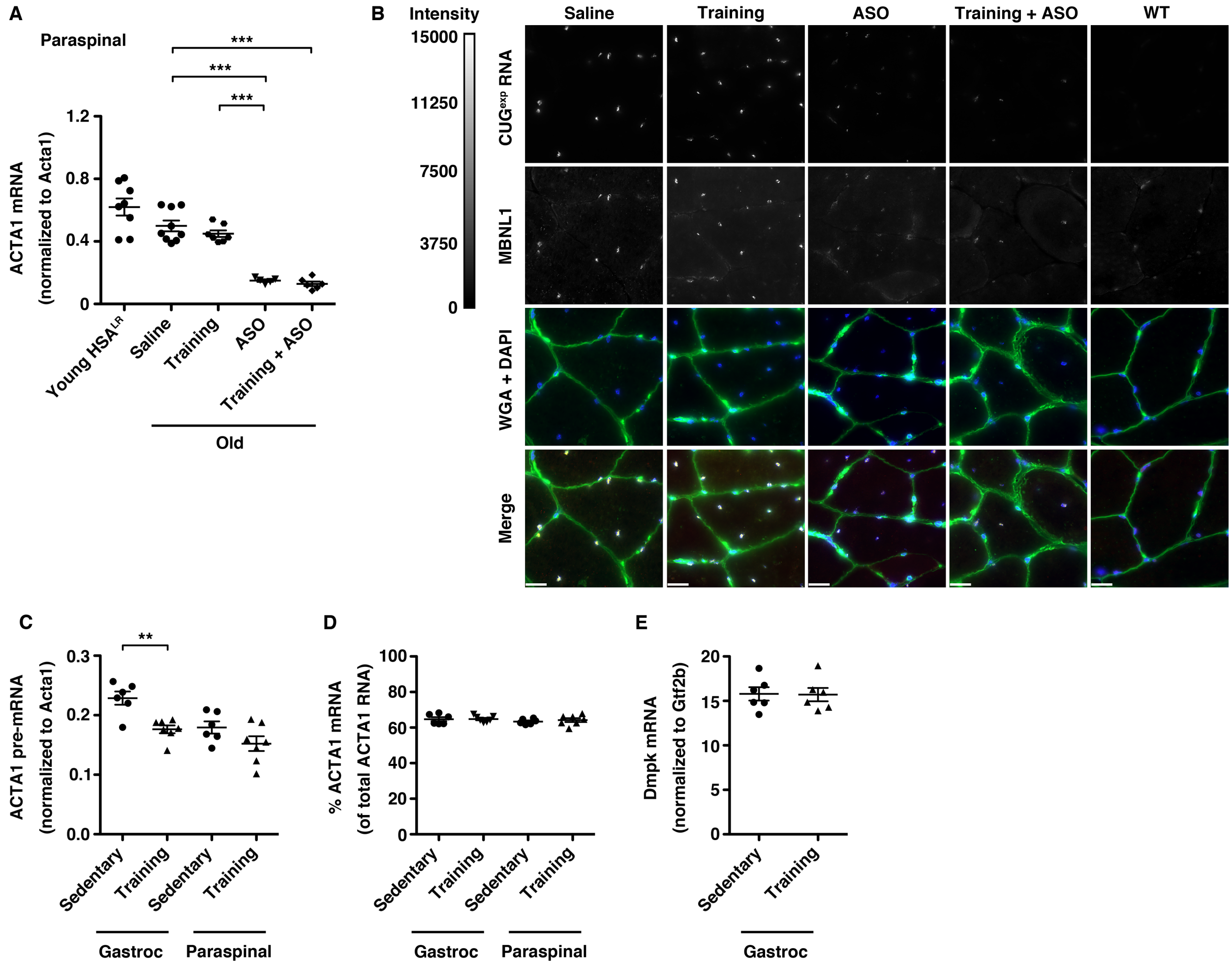

Figure S4. Molecular phenotype in muscles of DM1 mice with fatigue. We treated old HSA^{LR} mice (~ 14 months of age) with saline (N = 9), moderate intensity exercise training program (N = 7; Training; see Materials and Methods), ASO 445236 (N = 5), or a combination of training and ASO 445236 (N = 6) for 3 ½ months and analyzed at ~ 18 months of age. Young HSA^{LR} mice (2 - 4 months of age) (N = 8) served as controls. (A) Droplet digital PCR (ddPCR) quantification of *ACTA1*-CUG^{exp} transcripts in lumbar paraspinal muscles normalized to endogenous mouse *Acta1* transcripts. *** $P < 0.0001$; one-way ANOVA/Tukey. (B) Localization of CUG^{exp} RNA by fluorescence *in situ* hybridization (top row) and MBNL1 protein by immunofluorescence analysis (2nd row) in quadriceps muscles of experimental old HSA^{LR} mice and age-matched wild-type (WT) controls. Fluorescence intensity range is 0 - 15,000 grayscale units. Muscle fibers are highlighted by FITC-labeled wheat germ agglutinin (WGA; green) and nuclei by DAPI (blue). Bars = 20 μm. (C) To examine transcription of the *ACTA1* transgene, we used ddPCR to quantify *ACTA1* pre-mRNA normalized to mouse *Acta1* in gastrocnemius (gastroc) and lumbar paraspinal muscles of old mice that were sedentary (N = 6) or treated with an exercise training program (N = 7). ** $P < 0.001$; one-way ANOVA/Tukey. (D) ddPCR quantification of the percent of total *ACTA1* RNA that is mRNA in gastrocnemius and paraspinal muscles using primer probe sets specific for mRNA and pre-mRNA. The similarity of values suggests that the reduction of pre-mRNA in (B) and mRNA (Figure 5A) in mice that received exercise training is due reduced transcription of the *ACTA1*-CTG^{exp} transgene rather than enhanced metabolism of the CUG^{exp} mRNA in nuclear inclusions. (D) ddPCR quantification of *Dmpk* mRNA normalized to general transcription factor 2b (*Gtf2b*) in gastrocnemius muscle of old HSA^{LR} mice that were sedentary or treated with an exercise training regimen. Error bars indicate mean ± SEM. See also Figure 5.

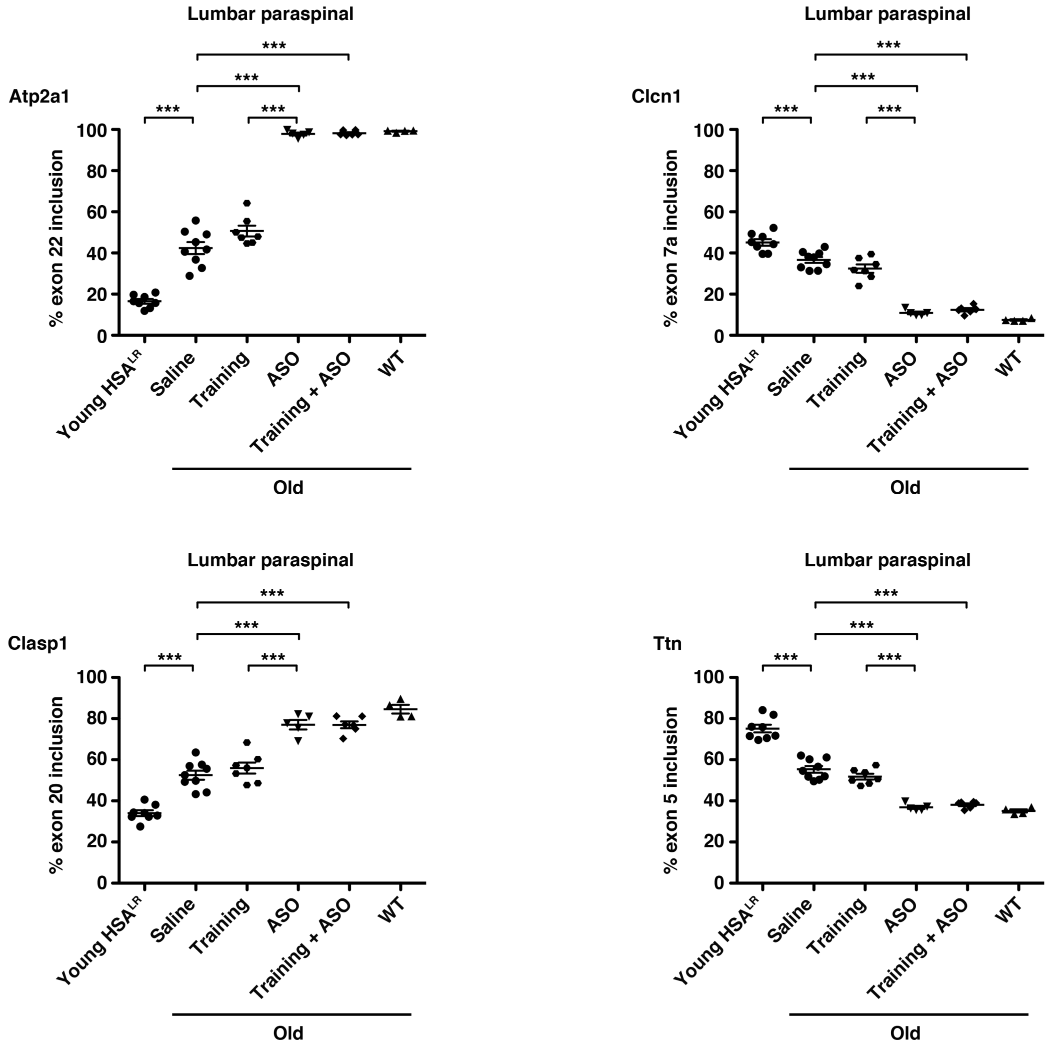

Figure S5. Alternative splicing in lumbar paraspinal muscles of DM1 mice with fatigue. We used RT-PCR and agarose gel electrophoresis to quantify alternative splicing of transcripts *Atp2a1* (upper left), *Clcn1* (upper right), *Clasp1* (lower left), and *Ttn* (lower right) in lumbar paraspinal muscles of each group. Old wild type (WT; ~ 18 months of age) (N = 4) served as additional controls. *** $P < 0.001$; one-way ANOVA/Tukey. Error bars indicate mean ± SEM. See also Figure 5.

CCACCGCAGCGGACAGCGCCAAGTGAAGCCTCGCTTCCCCTCCGCGGCGACCAGGGCCCGAGCCGAGAGTAGCAGTTGTAGCTACC
CGCCCAGgtagggcaggagttgggaggggacagggggacagggcactaccgaggggaacctgaaggactccggggcagaacccagt
cggttcacctggtcagccccaggcctgcgccctgagcgctgtgcctcgtctccggagccacacgcgctttaaaaaggaggcaagac
agtcagcctctggaaattagacttctccaaatttttctctagcccctttgggctcctttacctggcatgtaggatgtgcctaggga
gataaacggttttgctttagttgtcgccaaggcagttcccttccaaactagcgctagagcgaatgagcgagcagccaggaccaccc
attctgggtttccaacaggcgaaaaggccctttctgagtttgaaatgtcacagggttcctaacaggccactcttccctggatgggg
tgccaacgcctttcccatgggcatctccttccaccctcacgctggcccagcaagcaggcagtgctgaggccttatctccctaggtg
acagatgtggtcagggaggcgcagagaggatgggcactagcgtccagctcctggaacaggtgtcaggcagggagggcagacaggtc
ttggggaacatgttcccctggctatgtggacagaggacttctcagtggggtctcgcgaccctgtgccccttttcctggttcagggc
agcccttagccggggcaaaggtcgagaagagaacccctggtcgccgccctggcagaatttgagtggctccggcaggagatgtccct
aggttcctggggagggaggacgtcggggccagccaggcttaccccccctgccgctgagacttctgcgctgatgcaccgcgcctct
tcgcggtctccctgtccttgcagAAACTAGACACAATGTGCGACGAAGACGAGACCACCGCCCTCGTGTGCGACAATGGCTCCGGC
CTGGTGAAAGCCGGCTTCGCCGGGGATGACGCCCCTAGGGCCGTGTTCCCGTCCATCGTGGGCCGCCCCCGACACCAGgtcaggct
gcccctccgcagagggagccggctcggggtccccgcgtaagccagcctggtgccacccggagcggcgttaacgggtgcgtggtgtc
tcggctctgcagGGCGTCATGGTCGGTATGGGTCAGAAAGATTCCTACGTGGGCGACGAGGCTCAGAGCAAGAGAGGTATCCTGAC
CCTGAAGTACCCTATCGAGCACGGCATCATCACCAACTGGGATGACATGGAGAAGATCTGGCACCACACCTTCTACAACGAGCTTC
GCGTGGCTCCCGAGGAGCACCCCACCCTGCTCACCGAGGCCCCCCTCAATCCCAAGGCCAACCGCGAGAAGATGACCCAGATCATG
TTTGAGACCTTCAACGTGCCCGCCATGTACGTGGCCATCCAGGCCGTGCTGTCCCTCTACGCCTCCGGCAGGACCACCGgtgagtg
cccgctgcccccagtcccctctcccgcccccgcccccgcccccgcccccgccgctagcgctgagagcctagcctcggcctcgcccc
cagccactcactctctcccgcgcgcgcacagGCATCGTGCTGGACTCCGGCGACGGCGTCACCCACAACGTGCCCATTTATGAGGG
CTACGCGCTGCCGCACGCCATCATGCGCCTGGACCTGGCGGGCCGCGATCTCACCGACTACCTGATGAAGATCCTCACTGAGCGTG
GCTACTCCTTCGTGACCACAGgtgcgcggcgcccctgcaccccgggcgggagggccggcggcggcccctgagtgagggctcctctc
ctgcttctgccctccgcagCTGAGCGCGAGATCGTGCGCGACATCAAGGAGAAGCTGTGCTACGTGGCCCTGGACTTCGAGAACGA
GATGGCGACGGCCGCCTCCTCCTCCTCCCTGGAAAAGAGCTACGAGCTGCCAGACGGGCAGGTCATCACCATCGGCAACGAGCGCT
TCCGCTGCCCGGAGACGCTCTTCCAGCCCTCCTTCATCGgtgagccccgctcgccctcgccccggcccccaggccccgccggcccc
cggcgccccgagcttctgctcacgctccccgccgcggtccccagGTATGGAGTCGGCGGGCATTCACGAGACCACCTACAACAGCA
TCATGAAGTGTGACATCGACATCAGGAAGGACCTGTATGCCAACAACGTCATGTCGGGGGGCACCACGATGTACCCTGGGATCGCT
GACCGCATGCAGAAAGAGATCACCGCGCTGGCACCCAGCACCATGAAGATCAAGgtgggtggtggcctgcgcgggctgtcggcggg
gtgggctccagggtgaggtctccccacctcacgcgctgtcttgcagATCATCGCCCCGCCGGAGCGCAAATACTCGGTGTGGATCG
GCGGCTCCATCCTGGCCTCGCTGTCCACCTTCCAGCAGATGTGGATCACCAAGCAGGAGTACGACGAGGCCGGCCCTTCCATCGTC
CACCGCAAATGCTTCTAGACACACTCCACCTCCAGCACGCGACTTCTCAGGACGACGAATCTTCTCAATGGGGGGGCGGCTGAGCT
CCAGCCACCCCGCAGTCACTTTCTTTGTAACAACTTCCGT**TGCTGCCATCGTAAACTGAC**ACAGTGTTTATAACGTCTGCTGCTGC
TGCTGCTGCTGCTGCTGCTGCTGCTGCTGCTGCTGCTGCTGCTGCTGCTGCTGCTGCTGCTGCTGCTGCTGCTGCTGCTGCTGCTG
CTGCTGCTGCTGCTGCTGCTGCTGCTGCTGCTGCTGCTGCTGCTGCTGCTGCTGCTGCTGCTGCTGCTGCTGCTGCTGCTGCTGCT
GCTGCTGCTGCTGCTGCTGCTGCTGCTGCTGCTGCTGCTGCTGCTGCTGCTGCTGCTGCTGCTGCTGCTGCTGCTGCTGCTGCTGC
TGCTGCTGCTGCTGCTGCTGCTGCTGCTGCTGCTGCTGCTGCTGCTGCTGCTGCTGCTGCTGCTGCTGCTGCTGCTGCTGCTGCTG
CTGCTGCTGCTGCTGCTGCTGCTGCTGCTGCTGCTGCTGCTGCTGCTGCTGCTGCTGCTGCTGCTGCTGCTGCTGCTGCTGCTGCT
GCTGCTGCTGCTGCTGCTGCTGCTGCTGCTGCTGCTGCTGCTGCTGCTGCTGCTGCTGCTGCTGCTGCTGCTGCTGCTGCTGCTGC
TGCTGCTGCTGCTGCTGCTGCTGCTGCTGCTGCTGCTGCTGCTGCTGCTGCTGCTGCTGCTGCTGCTGCTGCTGCTGCTGCTGCTG
CTGCTGCTGCTGCTGCTGCTGCTGCTGCTGCTGCTGCTGCTGCTGCTGCTGCTGCTGCTGCTGCTGCTGCTGCTGCTGCTGCTGCT
GCTGCTGCTGCTGCTGCTGCTGCTGCTGCTGCTGCTGCTGCTGCTGGTACATACATTAACTTATTACCTCATTTTGTTAT
TTTTC**GAAACAAAGCCCTGTGGAAG**AAAATGGAAAACTTGAAGAAGCATTAAAGTCATTCTGTTAAGCTGCGT

Figure S6. *ACTA1* genomic sequence (UCSC genome browser) with 185 CTG repeats inserted into *Bsr* GI restriction site in the 3′ UTR that together comprise the transgene in the HSA^{LR} mouse model (see main text reference 29). Exon sequence is shown in upper case, intron sequence in lower case. To determine the CTG repeat length, we used a left primer that targets sequence 36 base pairs (bp) upstream of the CTG repeat sequence (turquiose background) and a right primer that targets sequence 59 bp down-stream of the CTG repeat sequence (yellow background). Therefore, the total sequence amplified outside of the CTG repeat is 36 bp + 59 bp = 95 bp. Based on an RT-PCR product size of approximately 650 bp evident in DNA from tail biopsy and gastrocnemius DNA (see Figure 7), the CTG repeat (underlined) in this model is approximately (650 bp - 95 bp non-CTG repeat sequence) = 555 bp. If the CTG repeat sequence = 555 bp, then the number of CTG repeats = 555/3 = approximately 185.

Table S1 ddPCR primer probe sets (PP sets). Probes for *ACTA1* mRNA and pre-mRNA, and mouse *Dmpk* mRNA were 5′-labeled with Fam, and 3′-labeled with Black Hole Quencher-1 (BHQ-1) (Biosearch Technologies). The amplicon size in base pairs (bp) is shown for each. We purchased an assay for mouse *Acta1* that uses a probe 5′-labeled with Hex and produces an amplicon of 117 bp (Bio-Rad; unique assay ID: dMmuCPE5088325).

PP set	Left primer (5′ - 3′)	Right primer (5′ - 3′)	Probe (5′ - 3′)	Amplicon size (bp)
ACTA1 mRNA	GTAGCTACCCGCCCAGAAACT	CCAGGCCGGAGCCATT	ACCACCGCCCTCGTGTGCG	83
ACTA1 pre-mRNA	ctgtccttgcagAAACTAGACAC	CCAGGCCGGAGCCATT	ACCACCGCCCTCGTGTGCG	79
mouse Dmpk mRNA	GACATATGCCAAGATTGTGCACTAC	CACGAATGAGGTCCTGAGCTT	AACACTTGTCGCTGCCGCTGGC	92

Table S2. PCR primers used to measure alternative splicing or estimate *ACTA1*-CTG^{exp} repeat length. PCR product size in base pairs (bp) with exon inclusion (+ ex) and exon exclusion (- ex) is shown.

Gene	Left primer (5′ - 3′)	Right primer (5′ - 3′)	Target exon(s)	+ ex size (bp)	- ex size (bp)
Atp2a1	GCTCATGGTCCTCAAGATCTCAC	GGGTCAGTGCCTCAGCTTTG	22	218	176
Clasp1	GTCGACGACAGGATCTCTCC	GAGCTCTGCCGTCTCGTG	20	198	174
Clcn1	TGAAGGAATACCTCACACTCAAGG	CACGGAACACAAAGGCACTG	7a	424	345
Titin	GTGTGAGTCGCTCCAGAAACG	CCACCACAGGACCATGTTATTTC	5	556	253
ACTA1-CTG	TGCTGCCATCGTAAACTGAC	CTTCCACAGGGCTTTGTTTC	CTG expansion	n/a	n/a

Movie S1. Monitoring spontaneous mouse movements in the x-, y-, and z-planes using an acrylic cage and infrared lasers. We tested all mice at night or synchronized to a reverse light cycle (shifted 12-hour:12-hour dark:light) room so that at the time of testing, the mice were behaviorally most active (see Materials and Methods). Representative monitoring is shown under red light.

Movie S2. Moderate intensity treadmill walking exercise. We exercised mice using a treadmill on a flat surface (no incline) at 11.5 m/min for 30 minutes, six days per week for 15 weeks (see Materials and Methods). Representative exercise is shown.
